# Single-cell sequencing dissects the transcriptional identity of activated fibroblasts and identifies novel persistent distal tubular injury patterns in kidney fibrosis

**DOI:** 10.1038/s41598-023-50195-0

**Published:** 2024-01-03

**Authors:** Valeria Rudman-Melnick, Mike Adam, Kaitlynn Stowers, Andrew Potter, Qing Ma, Saagar M. Chokshi, Davy Vanhoutte, Iñigo Valiente-Alandi, Diana M. Lindquist, Michelle L. Nieman, J. Matthew Kofron, Eunah Chung, Joo-Seop Park, S. Steven Potter, Prasad Devarajan

**Affiliations:** 1 Division of Nephrology and Hypertension, Cincinnati Children’s Hospital Medical Center, 3333 Burnet Avenue, Cincinnati, OH 45229-3039 USA; 2 Division Developmental Biology, Cincinnati Children’s Hospital Medical Center, Cincinnati, OH USA; 3 Division of Molecular Cardiovascular Biology, Cincinnati Children’s Hospital Medical Center, Cincinnati, OH USA; 4 Department of Pediatrics, University of Cincinnati, Cincinnati, OH USA; 5 Cytokinetics, San Francisco, CA USA; 6 Department of Radiology, University of Cincinnati, Cincinnati, OH USA; 7 Department of Radiology and Medical Imaging, Cincinnati Children’s Hospital Medical Center, Cincinnati, OH USA; 8 Department of Pharmacology and Systems Physiology, University of Cincinnati, Cincinnati, OH USA; 9 Division of Nephrology and Hypertension, Northwestern University Feinberg School of Medicine, Chicago, IL USA; 10 Feinberg Cardiovascular and Renal Research Institute, Northwestern University, Chicago, IL USA

**Keywords:** Computational biology and bioinformatics, Genetics, Molecular biology, Diseases, Nephrology

## Abstract

Examining kidney fibrosis is crucial for mechanistic understanding and developing targeted strategies against chronic kidney disease (CKD). Persistent fibroblast activation and tubular epithelial cell (TEC) injury are key CKD contributors. However, cellular and transcriptional landscapes of CKD and specific activated kidney fibroblast clusters remain elusive. Here, we analyzed single cell transcriptomic profiles of two clinically relevant kidney fibrosis models which induced robust kidney parenchymal remodeling. We dissected the molecular and cellular landscapes of kidney stroma and newly identified three distinctive fibroblast clusters with “secretory”, “contractile” and “vascular” transcriptional enrichments. Also, both injuries generated failed repair TECs (frTECs) characterized by decline of mature epithelial markers and elevation of stromal and injury markers. Notably, frTECs shared transcriptional identity with distal nephron segments of the embryonic kidney. Moreover, we identified that both models exhibited robust and previously unrecognized distal spatial pattern of TEC injury, outlined by persistent elevation of renal TEC injury markers including Krt8 and Vcam1, while the surviving proximal tubules (PTs) showed restored transcriptional signature. We also found that long-term kidney injuries activated a prominent nephrogenic signature, including *Sox4* and *Hox* gene elevation, which prevailed in the distal tubular segments. Our findings might advance understanding of and targeted intervention in fibrotic kidney disease.

## Introduction

Fibrosis is a key underlying process in CKD, resulting in a progressive functional decline with high prevalence, morbidity and mortality^1–3^. While early fibrotic response is essential for injury recovery^4^, excessive extracellular matrix (ECM) production leads to renal parenchymal fibrotic remodeling^5^. Since existing therapeutic options remain merely supportive^6^ and advanced CKD might result in end-stage kidney disease (ESKD) requiring lifelong dialysis or transplant^7^, mechanistic understanding of kidney fibrosis is paramount.

Aberrant injury induced fibroblast activation and appearance of myofibroblasts is a crucial pathologic contributor to kidney fibrosis^8–11^. Yet, existing approaches to ECM-producing renal cell population targeting remain controversial, due to the nonspecific expression of currently used markers such as *Acta2*, *Col1a1* and *Vim*^12–22^. A recent study examined human CKD and murine UUO induced renal fibrosis; however, defining kidney fibroblasts via ECM genes or *Pdgfrβ* might not allow for specific capturing of all heterogeneous stromal populations^12,23,24^.

Unresolved TEC injury and pro-fibrotic changes represent another key CKD landmark^25^. Multiple studies^26–30^ have examined healthy and abnormal PT transcriptional and translational profiles due to their crucial role in kidney metabolism and injury. Recent scRNA-seq analysis identified and pharmacologically tested molecular pathways involved in PT repair^31^. Also, several studies revealed new “repairing”, “injured” and “failed repair” PT states appearing during dynamic kidney injury response^32–34^. “Failed repair” PTs displayed dedifferentiated proinflammatory and profibrotic transcriptional states associated with CKD progression. However, little is known about the transcriptional signatures of distal nephron tubular segments, despite our recent report showing robust renal developmental program reactivation in distal segments^29^.

Here, we dissected the molecular and cellular events defining two clinically relevant murine models of kidney fibrosis. By combining multiple scRNA-seq replicate analysis with thorough validation, we identified three fibroblast clusters with distinctive transcriptional signatures along with persistent distal spatial pattern of long-term kidney tubular epithelial remodeling, injury and renal developmental program reactivation.

## Results

### Kidney fibrosis models exhibit proximal tubular loss, functional decline and novel cellular clusters

**Figure 1:**
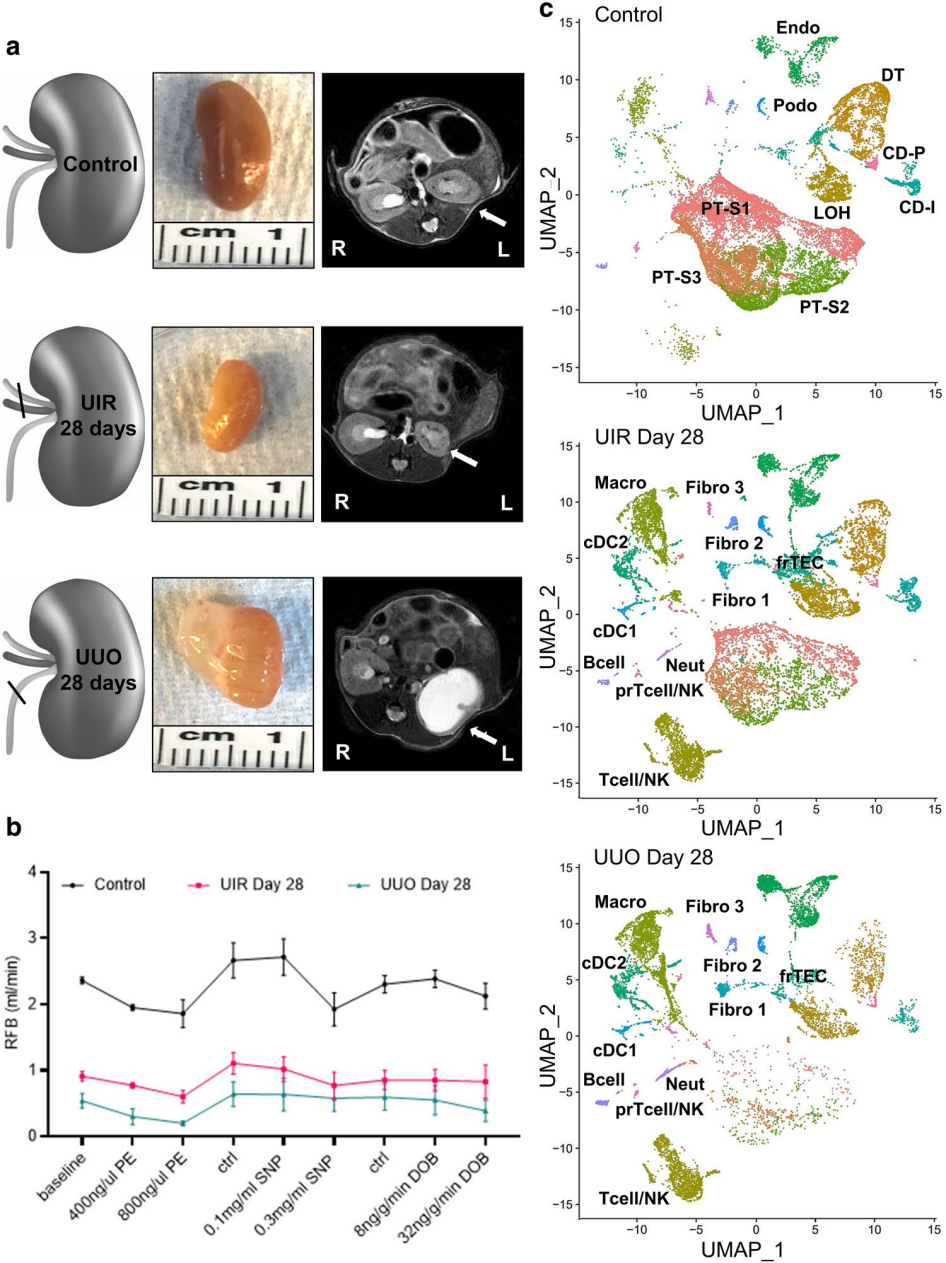
Ischemia/reperfusion and obstruction induced models of CKD elicit dramatic proximal tubular loss, kidney functional decline and novel cellular clusters. (**a**) Schemes of injury models (left), macroscopic (middle) and MRI (right) images of normal control, UIR and UUO day 28 kidneys. Kidneys are pointed to with white arrows. *R* right, *L* left. (**b**) Renal blood flow (RBF) at baseline, with vasoconstrictive (*PE* phenylephrine), vasodilative (*SNP* sodium nitroprusside) and inotropic agent (*DOB* dobutamine). Agents administered at 0.1 μl/min/gBW, *Ctrl* control interval between treatments, n = 3–4 per group, mean ± SD. **P ≤ 0.01, ***P ≤ 0.001, ****P ≤ 0.0001 compared to control, Student’s *t* test. (**c**) UMAPs show renal cell populations in the control, UIR and UUO kidneys (n = 3–5 per group). Clusters are distinguished by different colors. *PT* proximal tubules, *S1/2/3* segment 1/2/3, *LOH* loop of Henle, *DT* distal tubule, *CD-P* collecting duct principal, *CD-I* collecting duct intercalated, *Podo* podocytes, *Endo* endothelial, *Macro* macrophages, *Neutro* neutrophils, *cDC* conventional dendritic cells, *NK* natural killer, *prTcell* proliferating Tcell, *Fibro* fibroblasts, *frTEC* failed repair tubular epithelial cells.

**Figure 2:**
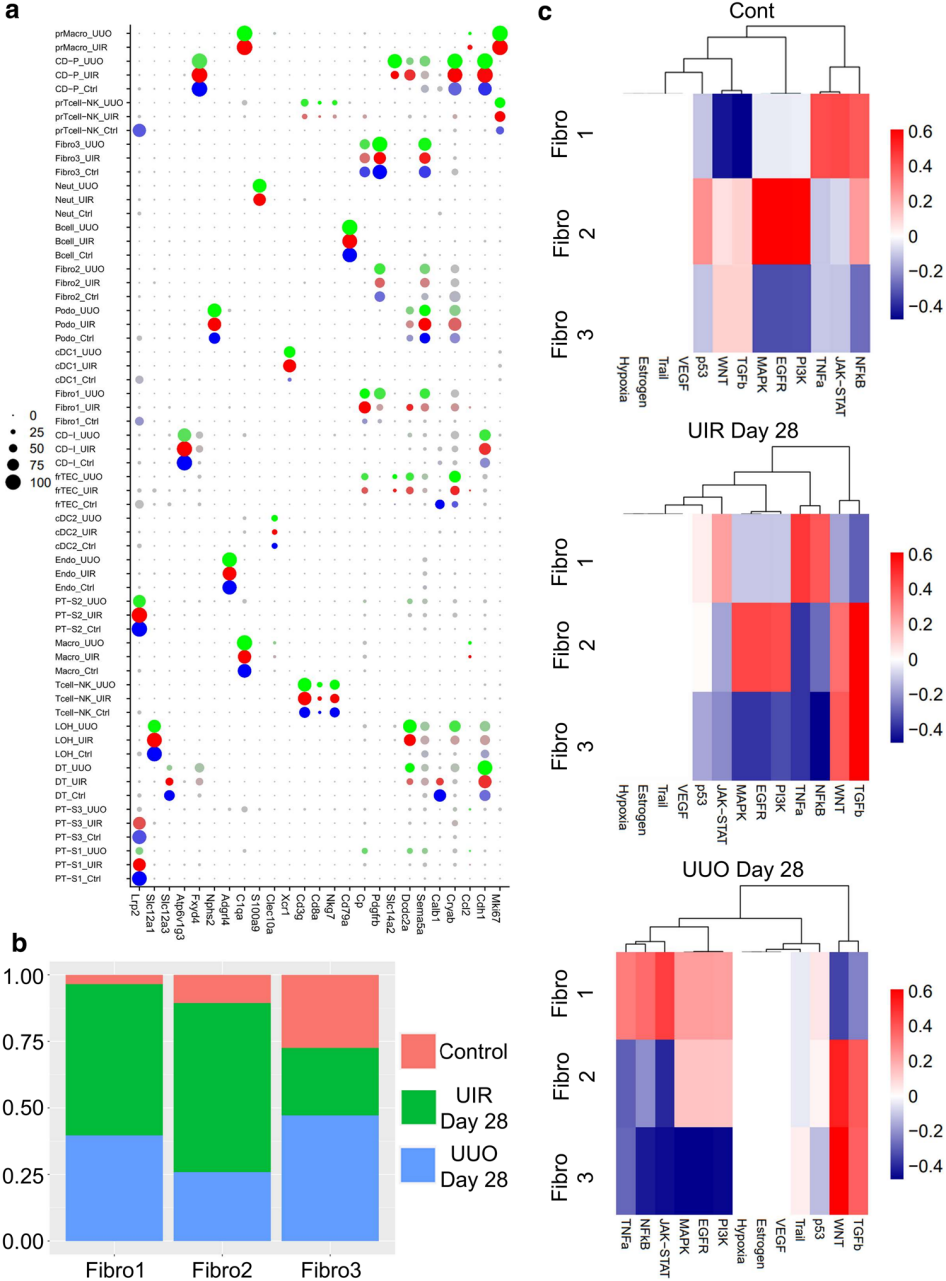
scRNA-seq dissects molecular and cell type proportion changes in the UIR and UUO models of renal fibrosis. (**a**) Dot plot of cell type-specific expression of marker genes for manually annotated clusters. Scale: dot size denotes percentage (0, 25, 50, 75, 100) of cells expressing the marker. Color intensity represents average gene expression values. Complete marker gene list is presented in Supplementary Table S1. (**b**) Relative fibroblast cluster proportion in the control (salmon), UIR (green) and UUO (blue) kidneys. Cell subset proportion change is shown relative to the listed conditions. (**c**) Pathway RespOnsive GENes (PROGENy) for activity inference analysis of three fibroblast clusters in the control, UIR and UUO kidneys. Complete PROGENy analysis of all populations is present in the Supplementary Fig. S24. Expression levels are represented with color gradient.

We established two clinically relevant kidney fibrosis models induced by UIR and UUO^35–37^. At day 28 post-injury, both models exhibited key CKD features including renal parenchymal loss and blood flow decline^38^ (Fig. 1a,b, Supplementary Fig. S1). Then, 10× Chromium scRNA-seq was carried out on the control, UIR and UUO kidney suspensions. Kidney cell populations were identified using unsupervised Uniform Manifold Approximation and Projection (UMAP) dimension reduction^39^, and datasets were cleansed of potential doublets, ambient RNA and cells with high mitochondrial and hemoglobin components (Supplementary Figs. S2–S4). Controls exhibited podocyte (*Nphs1/2*), endothelial (*Adgrl4*) and epithelial clusters, including large PT S1 (*Slc5a2/12*), S2 (*Cyp2e1/4b1, Slc22a6*) and S3 (*Acy3, Slc27a2*) subpopulations and distal nephron tubular segments, represented by loop of Henle (*Slc12a1*), distal tubule (*Slc12a3*), collecting duct principal (*Fxyd4*) and intercalated (*Atp6v1g3*) cells (Figs. 1c, 2a, Supplementary Fig. S5, Supplementary Table S1)^40^. UMAPs also revealed small myeloid clusters, including conventional dendritic (cDCs) 1 (*Xcir1, Clec9a*) and 2 (*Clec10a*)^41^, macrophages (*C1qa*) and neutrophils (*S100a9*), along with lymphoid B (*Cd79a*) and T/NK (*Cd3g, Cd8a, Nkg7*) cells^42^.

Both models caused remarkable cellular landscape changes, including dramatic PT decline (Fig. 1c, Supplementary Figs. S6 and S7a). We previously showed that AKI induced PT loss and dedifferentiation are restored to the mature gene expression as AKI resolves^29^. Instead, substantial S1–S3 PT decline persisted in our long-term models, indicating AKI-to-CKD progression. However, the surviving UIR and UUO Day 28 PTs returned to normal gene expression, except the UUO S3 segment which exhibited persistent low solute-linked carrier encoding gene levels and pro-inflammatory signature^33^ (*Lyz2, C1qa/b, Ifi30, S100a8/9*) (Supplementary Figs. S8 and S9). Moreover, scRNA-seq revealed a separate epithelial cluster, located between loop of Henle and distal tubule on the UMAP, which was present in the control and markedly expanded in the fibrotic kidneys (Fig. 1c, Supplementary Figs. S6 and S7a). We labeled them as “failed repair TECs” (frTECs) due to the simultaneous expression of epithelial (*Slc14a2, Cdh1, Calb1*), stromal (*Cp*) and injury related (*Cryab, Dcdc2a, Sema5a*) genes (Fig. 2a, Supplementary Figs. S5, S8 and S9).

Both models also exhibited evident inflammatory expansion, represented by increased myeloid and lymphoid infiltration along with proliferating macrophages (Figs. 1c, 2a, Supplementary Figs. S7b and S10a,b). While macrophages were the predominant immune fraction in the control, we observed Tcell/NK increase in the fibrotic kidneys. Moreover, scRNA-seq identified three separate fibroblast clusters, named “Fibro 1, 2 and 3” (Figs. 1c, 2b). We noted that Fibro 1 and 2 represented major stromal fractions of the fibrotic kidneys, while Fibro 3 cells were predominant in the controls (Supplementary Fig. S10). Validations on an independent control and injured mice cohorts verified substantial fibrotic remodeling, including statistically significant elevation of classical fibrosis markers Vim and αSma, inflammatory expansion and PT loss at the RNA and protein levels (Supplementary Figs. S11 and S12). Thus, we successfully established two models of kidney fibrosis exhibiting key CKD landmarks^43^.

### Prolonged kidney remodeling elicits pronounced epithelial-to-stromal crosstalk

Since our previous report showed enhanced cell-to-cell interactions in AKI^29^, we dissected the nature of intercellular crosstalk in advanced kidney injuries^44,45^ (Supplementary Table S2). Normal kidneys exhibited epithelial-to-epithelial, epithelial-to-stromal and epithelial-to-immune interactions via calmodulin, cadherin, G-protein coupled receptor, beta-2-microglobulin and urokinase pathways (Supplementary Figs. S13–S15). Both injury models caused enhanced communications (38,538 ligand-receptor pairs in UIR and 44,809 in UUO vs 24,984 pairs in control), including epithelial-to-stromal crosstalk (Supplementary Figs. S16–S19). scRNA-seq predicted that both injuries caused most notable increases in interactions between loop of Henle, collecting duct intercalated and PT S1 and 3 with Fibro 1 clusters, while the smallest number of receptor-ligand encoding gene pairs was found between collecting duct principal and stromal populations (Supplementary Fig. S20, Supplementary Table S3). TECs in the fibrotic kidneys interacted with stromal cells via collagen (*Col4a1-Cd47/Itgav/Itgb1*), osteopontin (*Spp1-Cd44/Itgav/Itgb1*), metalloproteinase 2 (*Timp2-Itgb1*), vascular cell adhesion molecule 1 (*Vcam1-Itgb1/b7/a4*) pathways. We also observed that *Col18a1*, which encodes the alpha chain of type XVIII collagen implicated in ureteric tree development^46^ and which we previously reported in AKI^29^, is elevated in the fibrotic kidney TECs, while it’s receptor encoding genes (*Gpc1/4, Itga5/b1*) are expressed in Fibro 1, 2 and 3 clusters, highlighting a putative interaction pathway present in both AKI and CKD models. scRNA-seq also showed that both models caused enhanced stromal-to-stromal and stromal-to-epithelial crosstalk (Supplementary Figs. S21 and S22). Fibroblasts interacted with each other and TECs via fibronectin, fibroblast growth factor 9/12, collagen, cadherin 1, transforming growth factor β (Tgfβ) and Wnt4 pathways. Of note, we revealed pronounced tubular-immune and stromal-immune communications, involving B cells, which is consistent with the recent report^42^.

### Advanced kidney remodeling exhibits three separate secretory, contractile and migratory fibroblast clusters with distinctive pathway enrichments

Using two independent scRNA-seq platforms, we repeatedly identified three distinctive fibroblast clusters in control and both fibrosis models (Fig. 1c, Supplementary Fig. S23), thus we further examined their unique transcriptional identities. Pathway responsive gene analysis (PROGENy)^47^ identified distinctions between Fibro 1, 2 and 3 clusters (Fig. 2c, Supplementary Fig. S24). Fibro 1 displayed elevation of TNFα, NFκB and JAK-STAT related genes while downregulating WNT and TGFβ. Fibro 2 was enriched for MAPK, EGFR and PI3K on the baseline and acquired WNT and TGFβ elevation in the injured kidneys. Fibro 3 exhibited WNT and TGFβ pathways enrichment on the baseline, which increased in both fibrosis models. Of note, cell division and death related p53 pathway was slightly upregulated in UIR and UUO Fibro 1 cluster and downregulated in Fibro 3 in both injured and control kidneys.

**Figure 3:**
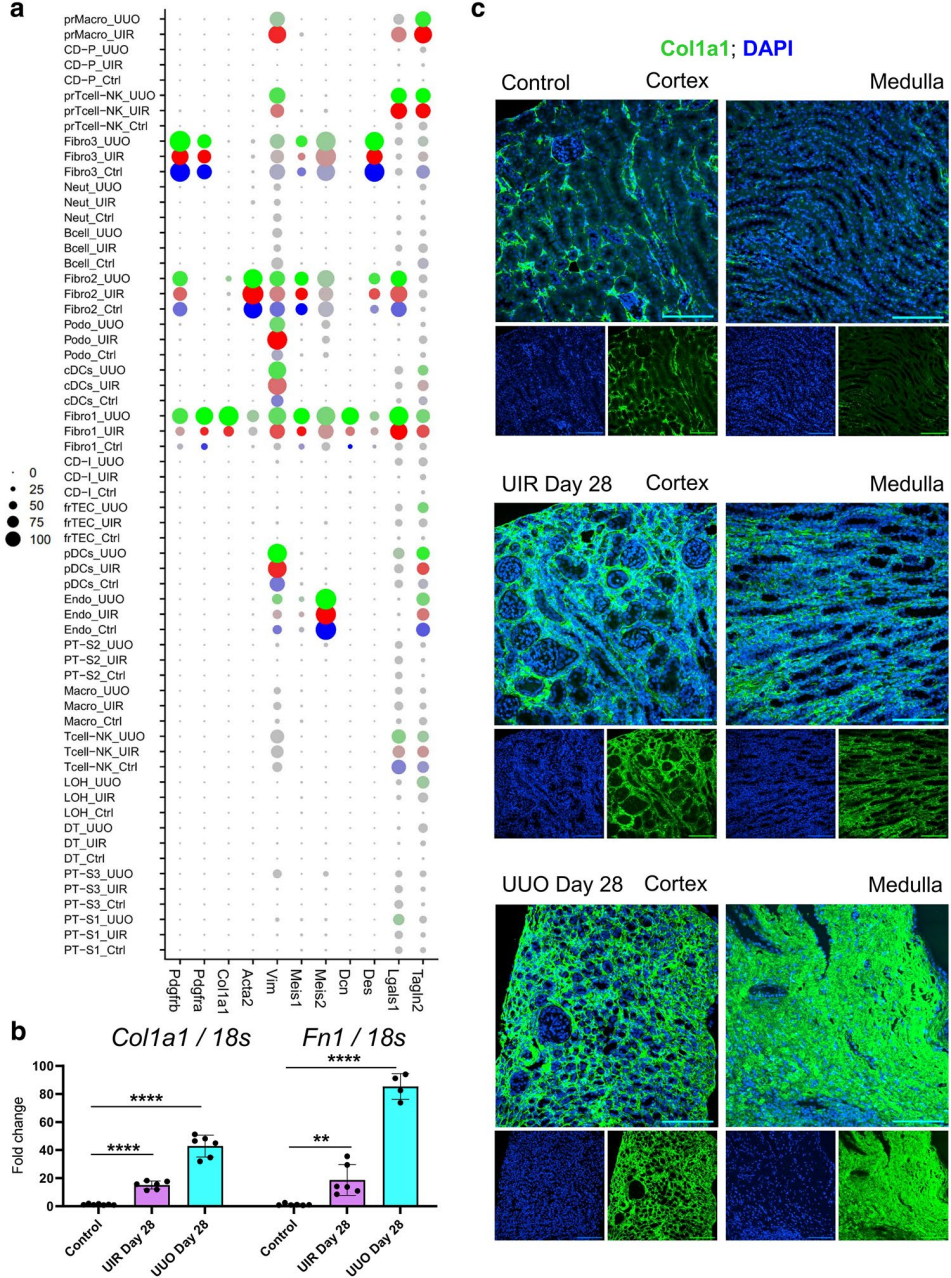
UIR and UUO elicit three transcriptionally distinctive fibroblast clusters. (**a**) Dot plot of cell type-specific expression of known fibrosis marker genes for manually annotated clusters. Scale: dot size denotes percentage (0, 25, 50, 75, 100) of cells expressing the marker. Color intensity represents average gene expression values. (**b**) qPCR of fibrosis (*Col1a1, Fn1*) markers, n = 4–7 per group. **P ≤ 0.01, ****P ≤ 0.0001 compared to control, Student’s *t* test. (**c**) Representative IF images of Col1a1 (green) and DAPI (blue) in control, UIR and UUO kidneys. Original magnification, × 40, maximal intensity projection, 0.40 μm/px zoom.

Comparative analysis showed that while all three fibroblast clusters expressed an established renal fibrosis marker *Pdgfbβ*^23^, each population elicited unique molecular identity. Specifically, genes enriched in Fibro 1, including *Col1a1, Fn1, Postn* and *Dcn*, were related to ECM production, cartilage development and ossification, which led us to labelling them as “secretory fibroblasts” (Fig. 3a, Supplementary Table S4). RT-qPCR on an independent murine control, UIR and UUO Day 28 cohorts verified scRNA-seq predicted upregulation of ECM related genes, such as *Col1a1* and *Fn1*, in a statistically significant manner (Fig. 3b). Cortical and medullary ECM deposition and fibrotic remodeling was also validated in both models by Picrosirius red staining (Supplementary Fig. S25a). Furthermore, immunofluorescence (IF) demonstrated remarkable elevation of Col1a1 cortical and medullary expression caused by both injuries in another validation experiment on an additional independently generated set of identical control, UIR and UUO animals (Fig. 3c). Thus, multiple rounds of independent validation reproducibly revealed the cortical and medullary spatial pattern of ECM deposition and fibrotic remodeling in both models.

**Figure 4:**
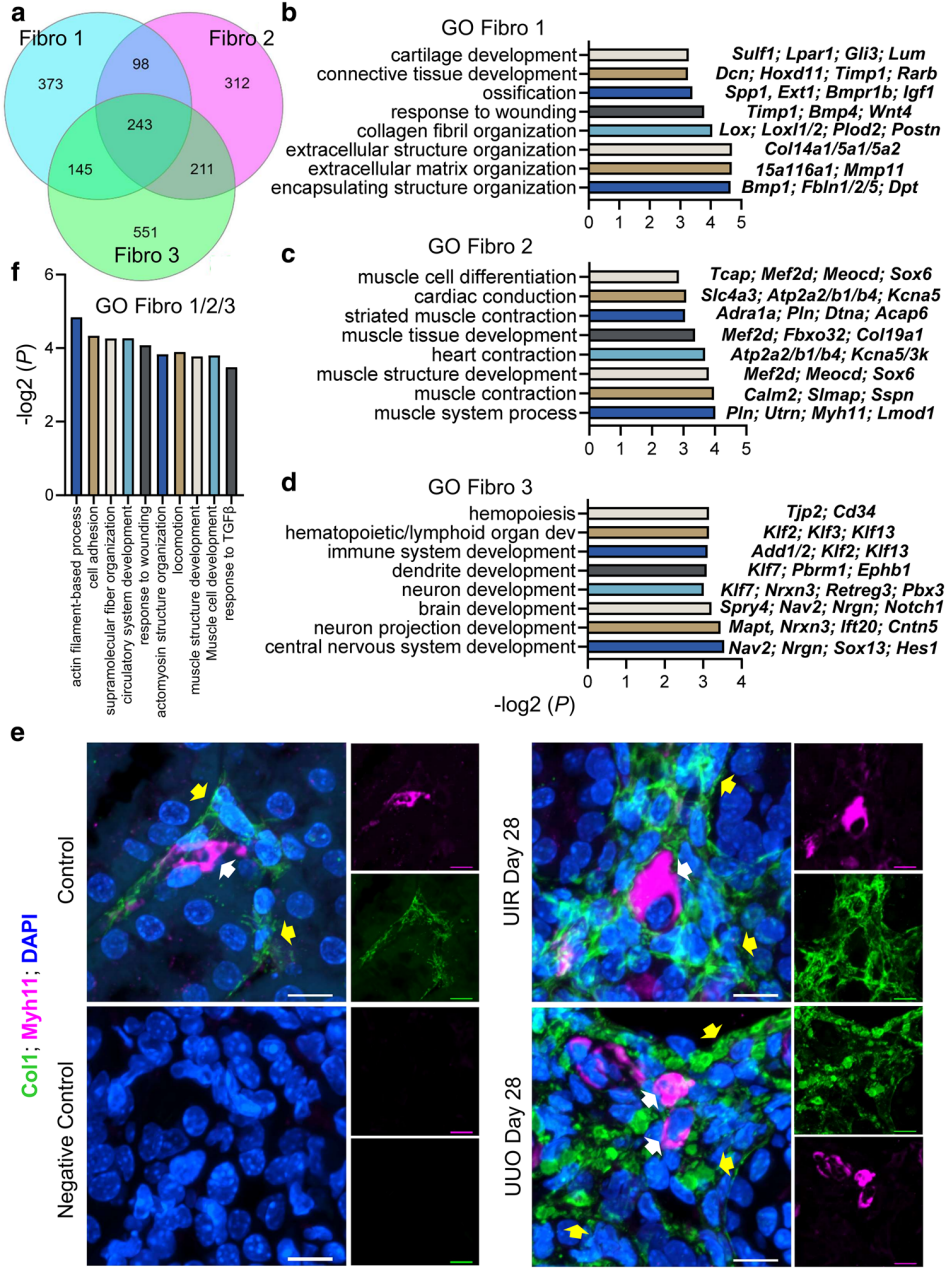
Three distinctive secretory, contractile and migratory fibroblast clusters emerge in advanced UIR and UUO. (**a**) Venn diagram displaying unique and shared Fibroblast 1, 2 and 3 marker genes. Complete lists of genes are presented in the Supplementary Table S4. (**b**–**d**) GO Biological process of fibroblast clusters unique marker genes vs other populations in control, UIR and UUO, − log2 (*P*). Representative genes are shown on the left for each biological process. Complete GO analysis is presented in the Supplementary Table S4. (**e**) Representative images of combined IF for Col1a1 (green), Myh11 (magenta) and DAPI (blue) in control, UIR and UUO kidneys. Original magnification, × 60, maximal intensity projection, 0.06 μm/px zoom. White arrows show Myh11-positive fibroblasts, yellow arrows show Col1a1-positive fibroblasts. (**f**) GO Biological process of fibroblast clusters 243 shared marker genes, − log2 (*P*). Complete GO analysis is presented in the Supplementary Table S4.

While “Fibro 1” populations exhibited pronounced ECM production and connective tissue development related signature, “Fibro 2” clusters were marked by muscle development, differentiation and contraction related genes, including historical myofibroblast marker *Acta2*; thus, we identified them as “contractile” (Figs. 3a, 4a–d). Since Fibro 3 signature was enriched for hemopoiesis and immune system related biological processes along with neuron projection and dendrite development, we called them “migratory”. Fibro 3 was also the predominant fibroblast type in the normal kidney and exhibited the smallest fold increase in UIR and UUO, while Fibro 1 cluster was the most abundant in both injuries, exhibiting remarkable increase in the fibrotic kidneys (Fig. 2b, Supplementary Fig. S10c). The existence of fibroblasts with distinctive molecular identities was validated by high resolution IF for Myh11, uniquely labelling Fibro 2 cells according to our scRNA-seq findings (Fig. 4c,e, Supplementary Table S4). We observed the population of Myh11-positive stromal cells the interstitial spaces of control and injured kidneys (Fig. 4d). These cells exhibited low to absent Col1a1 expression, coherent with scRNA-seq data showing very low *Col1a1* levels in “Fibro 2” (Fig. 3a). In the meantime, the surrounding Col1a1-enriched fibroblasts did not exhibit any Myh11 expression (Fig. 4d). These findings corroborate the scRNA-seq predicted molecular identities of healthy and fibrotic kidney stroma. In addition to the distinctions, three clusters had shared genes, related to TGFβ response, circulatory process and angiogenesis, cell migration and wound response^48^ (Fig. 4f, Supplementary Fig. S25b). However, many markers, previously used to identify fibroblasts, including *Vim, Lgals1, Tagln2* and *Meis1/2*, exhibited non-specific expression among off-target kidney populations (Fig. 3a).

### Long-term kidney injury induced frTECs share transcriptional identity with embryonic and adult distal nephron tubular segments

**Figure 5:**
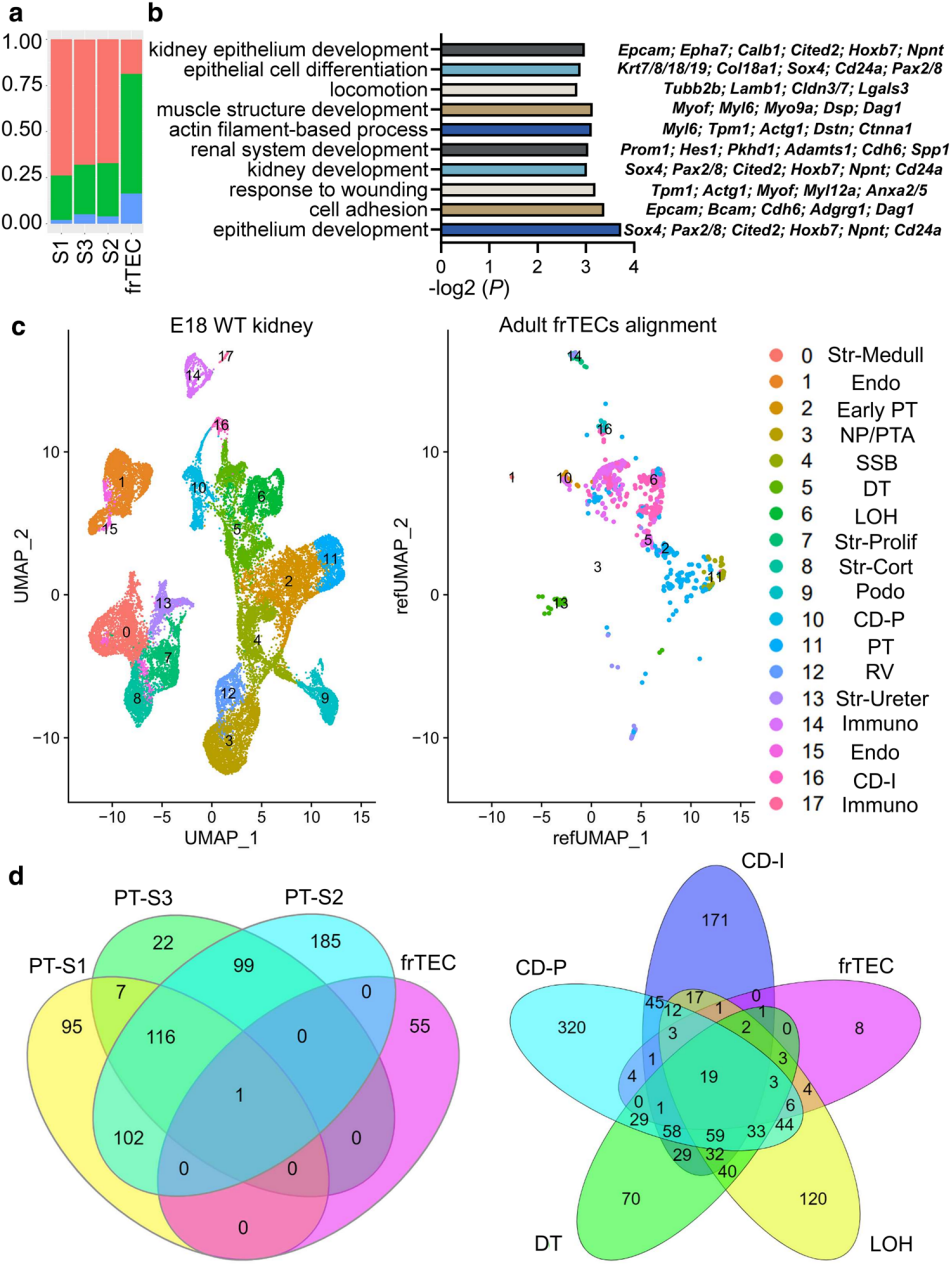
frTECs show transcriptional similarity with embryonic and adult distal segments of the nephron tubule. (**a**) Relative epithelial cluster proportion in the control (salmon), UIR (green) and UUO (blue) kidneys. Cell subset proportion change is shown relative to the listed conditions. (**b**) GO Biological process of “frTECs” marker genes vs other populations in control, UIR and UUO, − log2 (*P*). Names of the particular genes representing the biological process are listed on the left. Full biological process analysis and gene list is presented in the Supplementary Table S5. (**c**) UMAPs show renal cell populations in the E18 WT kidney (left), adult frTECs alignment to the E18 WT kidney populations (middle) and E18 WT kidney cluster designation (right). WT, wild type. Data source for E18 WT kidney scRNA-seq: GSE214024. Complete marker gene list for E18 WT kidney is presented in the Supplementary Table S6. (**d**) Venn diagrams displaying unique and shared PT S1–3, frTECs, loop of Henle, distal tubule and collecting duct principal and intercalated marker genes. Complete lists of genes are presented in the Supplementary Table S7.

Next, we questioned the transcriptional identity of tubular clusters in advanced kidney remodeling, particularly frTECs, which exhibited minimal presence in the normal kidney and expanded presence in the fibrotic kidneys (Figs. 1c and 5a). Gene ontology (GO)^49^ analysis identified that frTECs were enriched for kidney and epithelium development (*Pax2/8, Cited2, Sox4, Cd24a, Cdh6, Npnt*)^50–52^ (Fig. 5b, Supplementary Table S5), which indicates that these cells might be reverting to the dedifferentiated state as a result of injury^53^. Moreover, frTECs exhibited signs of mesenchymal transcriptional signature^25^, elevating genes related to locomotion, cell adhesion, muscle structure development and wounding. Since scRNA-seq demonstrated that frTECs are located between the distal segments of nephron tubule, including loop of Henle, distal tubule and collecting duct, and the fibroblast clusters on the UMAP (Fig. 1c), we asked what the cellular origins of this population might be. Bioinformatic comparison of our datasets to the previously generated E18 WT kidney scRNA-seq data (GSE214024) revealed that adult frTECs mainly align with embryonic loop of Henle, distal tubule and collecting duct (Fig. 5c, Supplementary Table S6). Moreover, comparison between adult tubular clusters produced substantial marker gene overlap between frTECs and distal nephron tubular segments, with only one gene common with S1–S3 PTs (Fig. 5d, Supplementary Table S7). Genes shared between frTECs, distal tubule, loop of Henle and collecting duct included kidney development (*Cd24a, Sox4, Pkhd1*)^51,52,54^, cell adhesion (*Epcam, Lgals3, Ezr, Dag1*) and apoptotic process (*Clu, Cldn7, S100a1*) related genes. Of note, frTECs did not exhibit increased *Ccl2*, which was previously shown to be upregulated in the PTs of sepsis-induced AKI^55^. scRNA-seq also revealed that control frTECs particularly elevated *Calb1*, which was also enriched in control distal tubules (Fig. 2a).

### Prolonged UIR and UUO exhibit persistent distal spatial patterns of tubular injury, while the remaining proximal tubules restore normal gene expression

**Figure 6:**
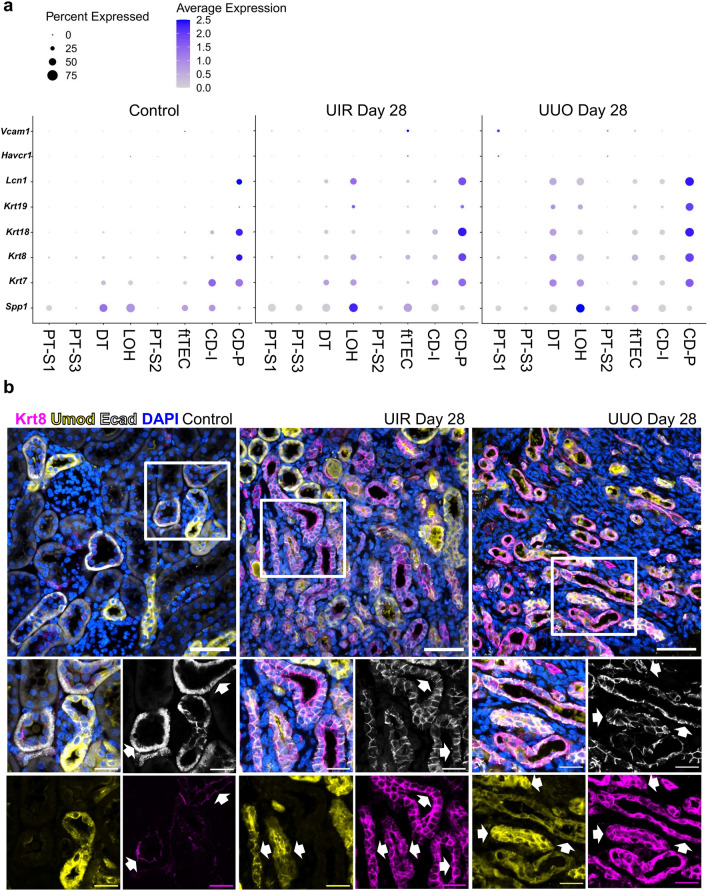
Long-term kidney parenchymal remodeling exhibits distal spatial pattern of tubular injury. (**a**) Dot plot of cell type-specific expression of renal epithelial injury markers for manually annotated clusters in the control, UIR and UUO kidney. Dot size denotes percentage of cells expressing the marker. Color intensity represents average gene expression values. (**b**) Upper panels—representative images of combined IF for Krt8 (magenta), Umod (yellow), Ecad (white) and DAPI (blue) in control, UIR and UUO kidneys. Original magnification, maximal intensity projection, × 60, 0.14 μm/px zoom. While frames indicate areas of magnification in the lower panels. Lower panels – representative images of combined IF for Krt8 (magenta), Umod (yellow), Ecad (white) and DAPI (blue) in control, UIR and UUO kidneys. Original magnification, × 60, maximal intensity projection, 0.09 μm/px zoom. White arrows indicate the areas of overlap between Krt8 and Umod/Ecad-expressing tubules.

We noted that frTECs and distal nephron tubular segments exhibited overlapping epithelial injury related molecular identity (Fig. 6a, Supplementary Table S7). This signature included the clinically recognized tubular injury marker *Lcn2*^56^ and other established markers of failed tubular repair, such as *Spp1*^57^ and *Krt7/8/18/*19^58^. This spatial tubular injury pattern was validated with IF on an independent control and fibrotic murine cohorts, which demonstrated increased Krt8 levels in E-cadherin (Ecad)-positive distal nephron tubular segments, including Uromodulin (Umod)-expressing loop of Henle of both UIR and UUO kidneys (Fig. 6b, Supplementary Fig. 26). While both scRNA-seq and IF showed moderate Krt8 expression only in parietal cells of the control collecting ducts, both models elicited dramatic overlap between Krt8 upregulation and Umod/Ecad-positive tubular segments. On the contrary, the remaining PTs restored normal mature gene expression signatures by Day 28 (Fig. 6a, Supplementary Figs. S5, S8 and S9). scRNA-seq revealed no elevation of *Havcr1*, an established marker of PT injury^59^, in any tubular clusters (Fig. 6a). Moreover, the surviving PTs were spared by other tubular injury markers, including Krt8, which was not present in the remaining Lotus tetragonolobus lectin (LTL)-positive PTs while labelling cortical and medullary Umod-expressing loop of Henle and Dolichos biflorus agglutinin (DBA)-positive distal tubule/collecting duct (Supplementary Fig. S27).

**Figure 7:**
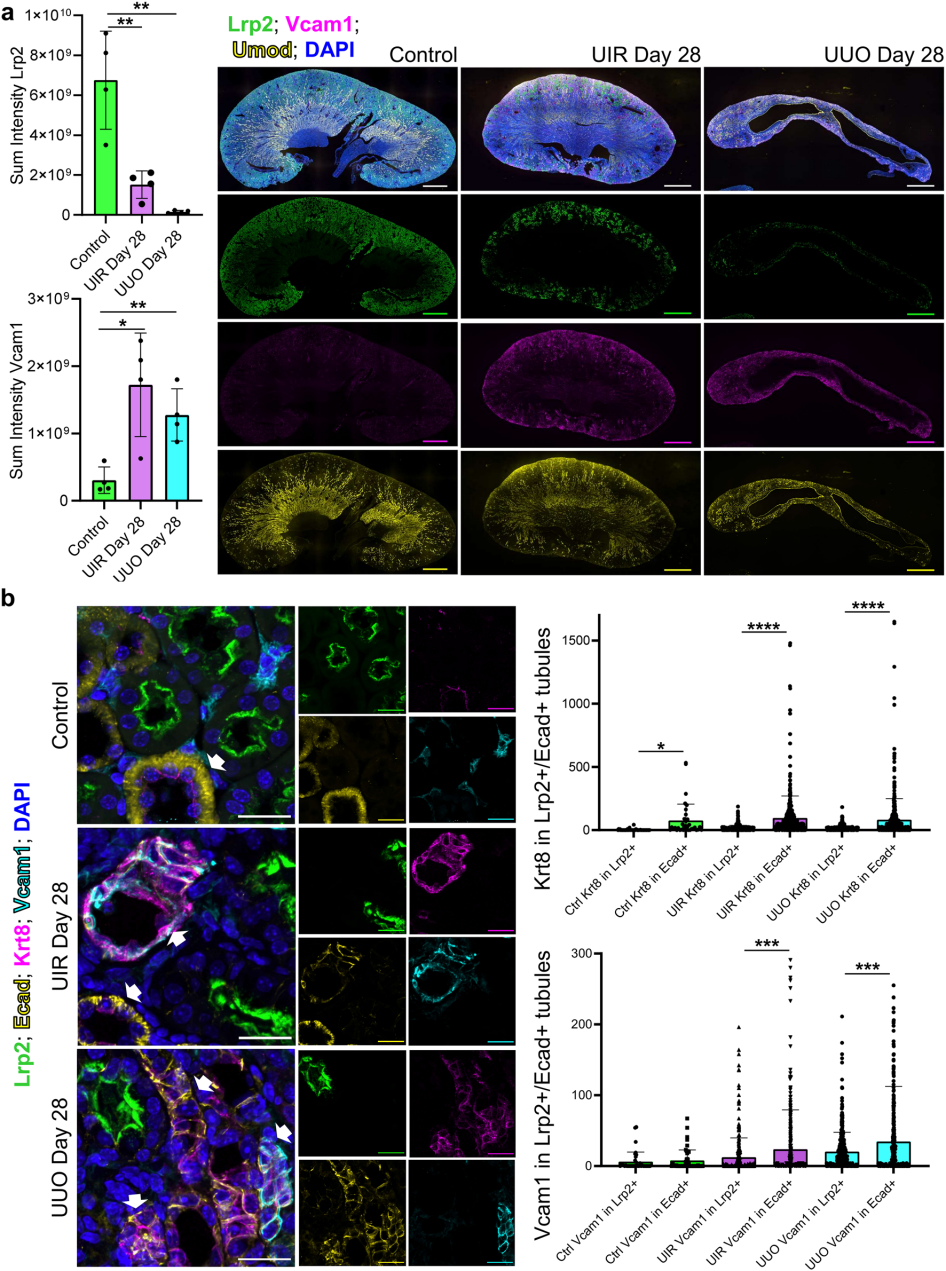
Loop of Henle and Krt8-positive segments of the nephron tubule exhibit persistent Vcam1 elevation at advanced kidney fibrotic remodeling stages. (**a**) Quantitative analysis (left) and whole-kidney representative images (right) of combined IF for Lrp2 (green), Umod (yellow), Vcam1 (magenta) and DAPI (blue) in the control, UIR and UUO kidneys. Original magnification, × 10, maximal intensity projection. Quantitative analysis: n = 4 per group, *P ≤ 0.05, **P ≤ 0.01, compared to control, Student’s *t* test. (**b**) Representative images (left) and quantitative analysis (right) of combined IF for Lrp2 (green), Ecad (yellow), Vcam1 (cyan), Krt8 (magenta) and DAPI (blue) in the control, UIR and UUO kidneys. Original magnification, × 60, maximal intensity projection, 0.28 μm/px zoom. White arrows highlight Vcam1 and Krt8 colocalization with Ecad. Quantitative analysis: n = 4 animals per group, 3–4 images per animal, ***P ≤ 0.001, ****P ≤ 0.0001, Student’s *t* test.

We noted that frTECs expressed several markers previously used to label maladaptively repaired PTs, including *Dcdc2a, Sema5a* and *Vcam1* (Figs. 2a, 6a)^33,60^. Quantitative whole-kidney IF analysis revealed that both UIR and UUO result in dramatic Lrp2-positive PT decline and Vcam1 elevation at Day 28 (Fig. 7a). We observed that while normal kidneys express Vcam1 in the interstitial spaces, both injuries resulted in elevated intratubular Vcam1, including colocalization with Krt8 (Fig. 7b, Supplementary Fig. S28). To further dissect the tubular injury patterns in kidney fibrosis, we performed quantitative IF analysis of Krt8 and Vcam1 expression in the proximal (Lrp2-positive) and distal (Ecad-positive) nephron tubular segments (Fig. 7b). As we (Fig. 2a) and others^61^ show, Ecad exhibits negligent proximal tubular expression while labelling loop of Henle, distal tubule and both intercalated and parietal cells of the collecting duct in control and fibrotic kidneys. Our quantitative analysis revealed that Ecad-expressing tubular segments exhibited remarkably higher Krt8 and Vcam1 levels compared to Lrp2-positive proximal tubules in both injuries. Overall, our data shows that distal segments of the nephron tubule endure persistent and unresolved injury in advanced kidney fibrotic remodeling.

### Kidney fibrosis causes renal developmental program reactivation in the stromal and distal nephron tubular populations

**Figure 8:**
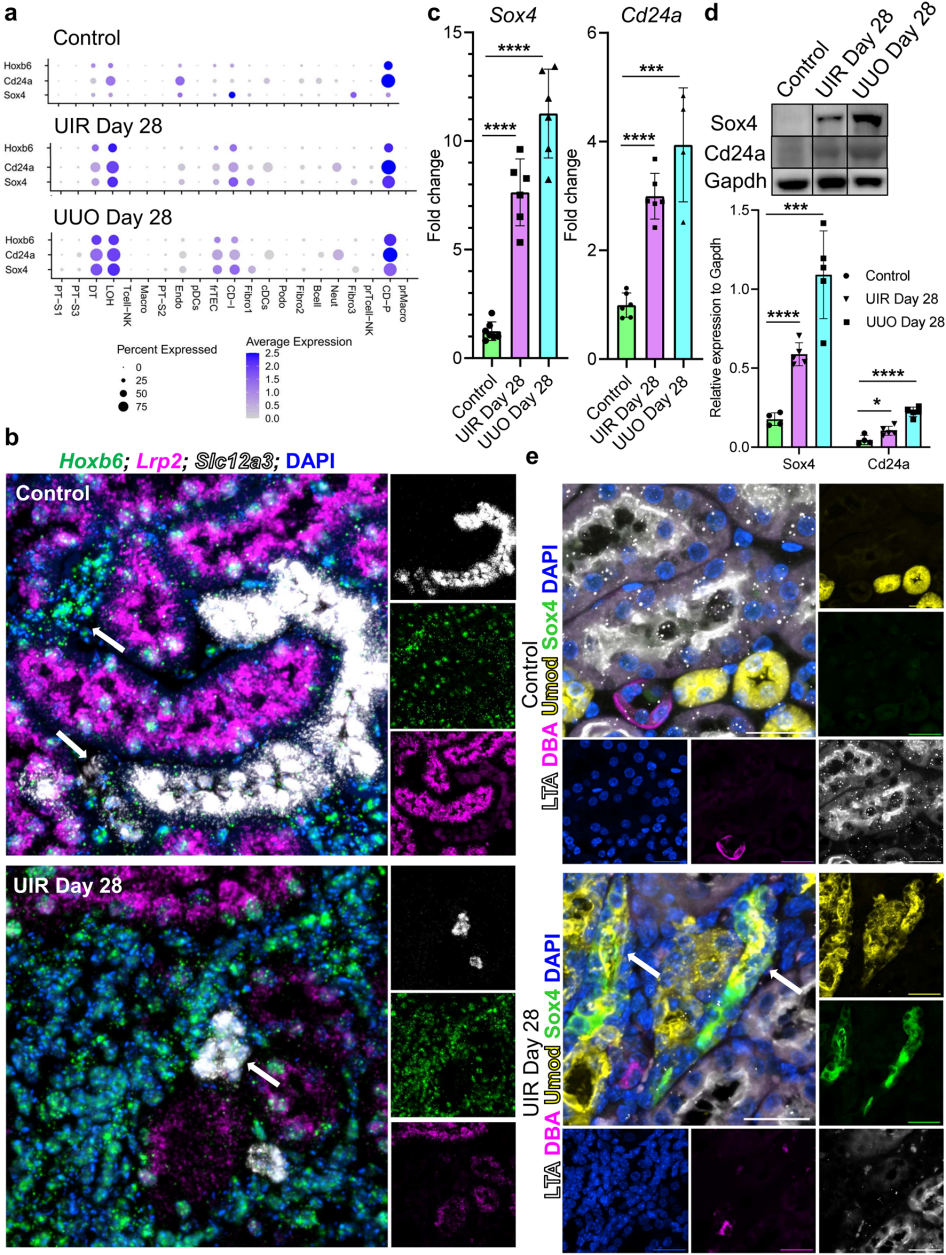
Advanced fibrotic injuries cause renal developmental program reactivation in the distal nephron tubular segments of adult kidney. (**a**) Dot plot of cell type-specific expression of *Hoxb6, Cd24a* and *Sox4* for manually annotated clusters in the control, UIR and UUO kidney. Dot size denotes percentage of cells expressing the marker. Color intensity represents average gene expression values. (**b**) Representative RNAscope images for *Hoxb6* (green), *Lrp2* (magenta), *Slc12a3* (white) and DAPI in the control and UIR kidneys. Original magnification, × 60, maximal intensity projection, 0.14 μm/px zoom. White arrows show *Hoxb6* expression in *Lrp2*-nehative and *Slc12a3*-positive tubules. (**c**) *Sox4* and *Cd24a* qPCR in control and fibrotic kidneys, n = 4–7 per group. (**d**) Representative bands and quantifications of Sox4 and Cd24a Western blots, n = 4–5 per group. Representative bands are cropped out of the original gels and are separated by the black border, the unprocessed original blots/gels are presented in Supplementary Fig. S29. *P ≤ 0.05, ***P ≤ 0.001, ****P ≤ 0.0001 compared to control, Student’s *t* test for (**c**) and (**d**). (**e**) Representative images of combined IF for Sox4 (green), Umod (yellow), DAB (magenta), LTL (white) and DAPI (blue), control and UIR kidneys. Original magnification, maximal intensity projection, × 60, 0.09 μm/px zoom. Areas of Sox4 co-localization with Umod are shown with white arrows.

Among the molecular changes underlying pathologic long-term kidney remodeling, we noted robust reactivation of genes normally expressed during nephrogenesis, including *Hox* genes (Fig. 8a, Supplementary Fig. S29)^62^. *Hoxd11* was elevated in Fibro 1 clusters of fibrotic kidneys, while some other isoforms including *Hoxb6* were upregulated in distal segments of the UIR and UUO nephron tubule, which was validated with RNAscope (Fig. 8b). Our data also showed elevation of renal developmental genes *Cd24a* and *Sox4* in both UIR and UUO. We recently reported that AKI elicited increased *Sox4* and Cd24a expression, which returned to baseline as the injury resolved^29^. However, scRNA-seq revealed persistent upregulation of these nephrogenic genes in both models of AKI-to-CKD transition, which was validated in additional UIR and UUO cohorts at the RNA and protein levels (Fig. 8c,d, Supplementary Fig. S30). Of note, both renal developmental genes were elevated in the distal nephron tubular segments, frTECs and fibroblasts, sparing the PTs. IF corroborated scRNA-seq findings, identifying that fibrosis caused Sox4 elevation in the loop of Henle, sparing the remaining LTL-positive PTs (Fig. 8e). Collectively, we revealed that AKI-to-CKD transition causes persistent nephrogenic signaling reactivation in multiple populations, including distal segments of the nephron tubule.

## Discussion

This study presents a single cell model-specific transcriptional profiling of fibrotic CKD. With combination of scRNA-seq and thorough validation, we reveal key cellular and molecular mechanisms of long-term kidney remodeling and a novel putative kidney fibroblast marker.

We previously created a thorough transcriptional profiling of AKI recovery^29^. The current study focuses on maladaptive long-term kidney injury response in two clinically relevant murine models of AKI-to-CKD transition. As our previous report suggests, first signs of kidney fibrosis and maladaptive responses develop on Day 14 after the injury induction. Thus, Day 28 was chosen for this study to ensure the complete onset of advanced fibrosis and kidney remodeling. UIR and UUO Day 28 repeatedly displayed key CKD features, such as kidney parenchymal reduction and functional blood flow decline. scRNA-seq performed on multiple replicates with two independent platforms showed dramatic PT loss, inflammatory infiltration, and stromal expansion in both models, which was validated in separate control, UIR and UUO cohorts.

Both models elicited three novel fibroblast clusters, consistent with recent reports revealing kidney stroma heterogeneity^63^. Particularly, the recent study by Kuppe et al.^23^ used sorting to isolate PT and non-PT fractions from hypertensive CKD patients and dissect the heterogeneity of renal interstitium. Consistent with their human findings, we identified that murine CKD results in higher ECM related gene expression compared to the control. We found that while both UIR and UUO induce crucial CKD pathological landmarks, UUO causes more robust renal blood flow decline, tubular injury, inflammation and epithelial parenchymal remodeling. Thus, we established two independent models allowing to simultaneously examine the molecular and cellular changes in the fibrotic kidney with respect to the injury cause and severity. Among the non-PT fraction, Kuppe et al. identified the mesenchymal populations, including *Postn*-myofibroblasts, *Dcn*-positive fibroblasts and *Cox4i2*-positive pericytes, all exhibiting high ECM related gene expression score. Consistent with that, we observed ECM and collagen fiber organization related gene enrichment in the Fibro 1 population, thus annotating it as the most responsible for fibrotic remodeling. However, we observed that while all three of scRNA-seq identified fibroblast clusters expressed *Pdgfrβ*, Fibro 1 was the only fibroblast fraction labelled by *Dcn* and *Col1a1*. Thus, our data suggests that those ECM related genes might not be used to comprehensively label kidney fibroblasts. On the contrary, Fibro 2 was the only population which elevated classic myofibroblast marker *Acta2*, thus we labelled them as contractile. We noted that Fibro 3 elevated pericyte markers^64^ *Pdgfrβ* and *Dsm* relative to other fibroblasts. Moreover, control, UIR and UUO Fibro 3 exhibited increased *Pdgfrα* recently implicated in vascular fibrosis^65^. While Fibro 1 and 2 clusters were expanded in UIR and UUO compared to the control, Fibro 3 represented major stromal fraction in the normal kidney. Overall, our findings contribute to understanding the heterogeneity of kidney stroma and highlight the need for a specific marker which would allow for thorough labeling and targeting of all activated kidney fibroblasts with no off-target expression.

We also show that both models cause significant PT dropout compared to the control, with UUO causing near-total PT loss and more aggressive fibrosis than UIR, which is consistent with a recent report^63^ which also identified diverse PT injury states and repair outcomes: UUO Day 14 elicited large aberrantly repaired PT fraction and persistent healthy PTs decline, while UIR Day 28 exhibited near-total repair. Instead, we found that both UIR and UUO at Day 28 exhibit persistent PT decline. This divergence in the UIR response might reflect the differential effects of ischemia duration on the PT injury.

We also found that despite the substantial overall dropout, the remaining PTs displayed largely normal gene expression. The observed lack of *Havcr1* at UIR and UUO Day 28 might be explained by earlier loss of maladaptively repaired PTs along with mature gene expression restoration in the surviving ones. The enduring distal tubular segment injury was indicated by near-total overlap between an established epithelial injury marker Krt8 and distal nephron tubular markers Umod and Ecad. This pattern was further validated with quantitative IF analysis, revealing significantly elevated tubular injury markers Krt8 and Vcam1 in Ecad-positive distal nephron tubular segments compared to proximal tubules in both fibrosis models. Moreover, frTECs exhibited transcriptional similarity with embryonic and adult distal segments of the nephron tubule on the marker gene level, including *Calb1, Slc14a2* and *Cdh1*, showing little transcriptional overlap with PTs. The divergence between our observation and other reports showing predominantly proximal origin of maladaptively repaired tubules might be explained by distinctive origins of “frTECs” at different injury stages. Some of the observed difference might also originate from intentional focusing on PTs or even enriching them in the final datasets via sorting^23,32,33^, which might result in distal nephron tubular injury being out of focus. Further fate tracing studies using transgenic reporter lines specifically labeling proximal and distal nephron tubular segments will help to reveal the origin of frTECs at advanced kidney injury stages. Of note, we found that *Sox4*, recently reported in the human AKI urine using scRNA-seq^66^, was strongly elevated in UIR and UUO loop of Henle, distal tubule and principal cells. Since embryonic *Sox4* ablation caused accelerated early-onset CKD and ESKD, targeting this newly identified injury induced nephrogenic signature might offer a promising strategy in intercepting adult AKI-to-CKD transition. Our findings highlight the previously unrecognized salutary response of the distal nephron in kidney fibrosis, which may be targeted for diagnostic and therapeutic interventions.

## Methods

### Animals

The Institutional Care and Use Committee (IACUC) of Cincinnati Children’s Hospital Medical Center approved all animal procedures in the study. All the experiments and methods, including animal husbandry and monitoring, were performed in accordance with relevant IACUC guidelines and regulations. Data reporting in the manuscript follows the recommendations in the ARRIVE guidelines^67^. Unilateral ischemia/reperfusion (UIR) was induced via atraumatic left renal pedicle clamping for 30 min at 37 °C and unilateral ureter obstruction (UUO) was induced via irreversible left ureter ligation in 10 weeks old male C57Bl/6 mice (n = 3 for 10× Chromium scRNA-seq, 1 for Drop-seq per model). Mice were anesthetized by 3% Isoflurane anesthesia gas before the procedures and received 1.5–2% Isoflurane anesthesia gas during operating. Buprenorphine sustained release (SR) was administered after operating 0.5–1 mg/kg subcutaneously. The kidneys were harvested at Day 28 post-injury. For euthanasia, mice were exposed to overdose inhalant anesthetic (Isoflurane), followed by exsanguination and organ harvest. Naive mice of the same age, strain and sex (n = 5) were used as controls.

### Sample preparation and scRNA-seq analysis

Single cell suspensions were prepared with *Bacillus licheniformis* cold active protease^68^ and sequenced using an Illumina Novaseq 6000 following the 10× Genomics protocol for library construction using the Single Cell 3′v3 chemistry. The fastq files were processed using 10× Genomics Cell Ranger v6.1.2 and ambient RNA was mediated by using the decontX function within the celda package^69^. Resulting datasets were further cleaned using doubletFinder package^70^ with 7.5% doublet occurrence per data set. For details, see the Supplemental Methods.

### Validation

Two independent control and injury induced C57Bl/C murine cohorts of identical age, sex and surgical treatment were used to validate the scRNA-seq findings (n = 4–7 per group).

### Equipment and settings

Macroscopic kidney images were obtained on Zeiss Axiovert 25 wide-field microscope. Magnetic resonance imaging (MRI) was performed using a horizontal 7 T Biospec MRI system (Bruker, Billerica, MA); axial images were acquired using a fast spin echo sequence with a repetition time of 2500 ms, echo time of 40.2 ms, echo train length of 16, 4 averages, 32 mm × 32 mm field of view, and an acquisition matrix of 200 × 200. IF images were produced on Nikon Ti-E AXR HD confocal with the resonant scanner, processed with NIS-Elements AR 5.2.00 artificial intelligence denoise algorithm and analyzed with Bitplane Imaris 10.0.0^29^. For Krt8 and Vcam1 IF quantitative analysis, 3–4 Z-stack images per animal (n = 4 animals per control, UIR Day 28 and UUO Day 28) were obtained at 0.28 μm/px resolution in the outer and inner cortex where proximal (Lrp2+) and distal (Ecad+) nephron tubular segments are present. Imaris 10.0.0 algorithm “Surfaces” was used to designate Lrp2- and Ecad-expressing tubules. The algorithm “Spots” was used to label Krt8 and Vcam1 expression and quantitatively assess it inside Lrp2+ versus Ecad+ tubules. Identical intensity settings were used to create Krt8 and Vcam1 spots across all experimental groups. The resulting Krt8 and Vcam1 expression for all analyzed tubules is presented in Fig. 7b. All images within an experimental group were obtained and displayed with the same optical configurations. Western blot imaging was performed using a ChemiDoc imaging system and Bio-Rad’s Image Lab Touch Software, quantitative analysis was done in ImageJ. For details on the used antibodies, including Sox4^71,72^ and Cd24a^29,73^, see the Supplemental Methods.

### Statistical analysis

scRNA-seq was reproduced in three independent runs using DropSeq and 10× Chromium platforms; validation was performed on leftover scRNA-seq suspensions and separate UIR, UUO and control mice (n = 4–6 per group). *P* values were generated using Student’s *t* test with *P < 0.05 representing the statistically significant difference. Data are presented as individual values, mean ± SD.

### Supplementary Information

Supplementary Information.

Supplementary Table S1.

Supplementary Table S2.

Supplementary Table S3.

Supplementary Table S4.

Supplementary Table S5.

Supplementary Table S6.

Supplementary Table S7.

## Data Availability

The datasets generated and/or analyzed during the current study are available at the Gene Expression Omnibus under accession number GSE198621.
