# Supplementary material for: Single-cell sequencing dissects the transcriptional identity of activated fibroblasts and identifies novel persistent distal tubular injury patterns in kidney fibrosis
Source: Sci Rep. 2024 Jan 3;14:439. doi: 10.1038/s41598-023-50195-0 (PMC10764314; doi:10.1038/s41598-023-50195-0)
Supplement: Supplementary file 1 — Supplementary Information. [file 41598_2023_50195_MOESM1_ESM.pdf]

Supplemental Material for

Single-cell sequencing dissects the transcriptional identity of activated fibroblasts and identifies novel persistent distal tubular injury patterns in kidney fibrosis

Valeria Rudman-Melnick[1], Mike Adam[2], Kaitlynn Stowers[2], Andrew Potter[2], Qing Ma[1], Saagar M. Chokshi[1], Davy Vanhoutte[3,4], Iñigo Valiente-Alandi[5], Diana M. Lindquist[4,6,7], Michelle L. Nieman[8], J. Matthew Kofron[2,4], Eunah Chung[9], Joo-Seop Park[9], S. Steven Potter[2,4] and Prasad Devarajan[1,4]

[1]Division of Nephrology and Hypertension, Cincinnati Children's Hospital Medical Center, Cincinnati, OH, USA

[2]Division Developmental Biology, Cincinnati Children's Hospital Medical Center, Cincinnati, OH, USA

[3]Division of Molecular Cardiovascular Biology, Cincinnati Children's Hospital Medical Center, Cincinnati, OH, USA

[4]Department of Pediatrics, University of Cincinnati, OH, USA

[5]Cytokinetics, San Francisco, CA, USA

[6]Department of Radiology, University of Cincinnati, OH, USA

[7]Department of Radiology and Medical Imaging, Cincinnati Children's Hospital Medical Center, Cincinnati, OH, USA

[8]Department of Pharmacology and Systems Physiology, University of Cincinnati, OH, USA

[9]Feinberg Cardiovascular and Renal Research Institute, Northwestern University, IL, USA

Corresponding author: Prasad Devarajan, Division of Nephrology and Hypertension, Cincinnati Children's Hospital Medical Center, 3333 Burnet Avenue, Cincinnati, OH 45229-3039, USA, 513-636-4531, prasad.devarajan@cchmc.org

Supplemental Material List

Supplemental Methods

Supplemental Tables List and Legends

Supplemental Figures and Legends

Figure S1. Invasive hemodynamic reveals no significant systemic cardiovascular function changes caused by renal fibrosis models compared to the control.

Figure S2. Quality control cutoffs used on scRNA-seq data processing.

Figure S3. DoubletFinder package is used to identify the potential doublets in scRNA-seq data.

Figure S4. Gene distribution per condition in the control, UIR and UUO scRNA-seq data.

Figure S5. scRNA-seq identifies cellular landscape of the control kidneys.

Figure S6. scRNA-seq identifies the fractional composition of cellular clusters in the control, UIR and UUO Day 28 kidneys.

Figure S7. scRNA-seq dissects relative abundance of tubular epithelial and immune populations in the control and fibrotic kidneys.

Figure S8. scRNA-seq identifies cellular landscape of the UIR kidneys.

Figure S9. scRNA-seq identifies cellular landscape of the UUO kidneys.

Figure S10. scRNA-seq dissects relative abundance of immune and fibroblast populations in the control and fibrotic kidneys.

Figure S11. Validation corroborates UIR and UUO induced proximal tubule loss, fibroblast activation and inflammatory infiltration demonstrated by scRNA-seq.

Figure S12. Original uncropped blots for Figure S11b.

Figures S13-15. scRNA-seq dissects the molecular and cellular nature of the cell-to-cell crosstalk in the normal kidney.

Figures S16-19. scRNA-seq dissects the molecular and cellular nature of epithelial-to-stromal crosstalk in the fibrotic kidney.

Figure S20. Quantitative analysis reveals that both kidney fibrosis models cause enhanced distal nephron tubule segment to stromal interactions.

Figures S21 and S22. scRNA-seq dissects the molecular nature of the crosstalk between fibroblast clusters in the advanced kidney injury.

Figure S23. Drop-seq analysis corroborates 10x Chromium scRNA-seq identified three distinctive fibroblast clusters present in both UIR and UUO Day 28.

Figure S24. PROGENy analysis reveals the molecular signaling pathway changes caused in the kidney cell populations by long-term fibrotic injuries.

Figure S25. Picrosirius Red staining validates ECM deposition in both cortex and medulla of UIR and UUO treated kidneys compared to the control.

Figure S25. Picrosirius Red staining validates ECM deposition in both cortex and medulla of UIR and UUO treated kidneys compared to the control.

Figure S26. Long-term kidney parenchymal remodeling exhibits distal spatial pattern of

tubular injury.

Figure S27. Krt8 expression spares LTL-positive proximal tubules and overlaps with distal nephron tubular segments.

Figure S28. UIR and UUO induced kidney fibrosis caused intratubular Vcam1 expression in Umod- and Krt8-positive tubules.

Figure S29. Advanced fibrotic injuries cause renal developmental program reactivation in the distal nephron tubular segments of adult kidney.

Figure S30. Original uncropped blots for Figure 8c.

Supplemental Methods

scRNA-seq procedure. UIR, UUO and control mice were intraperitoneally injected with 100 μL heparin (100 U/mL), anesthetized with isoflurane chamber and euthanized via exsanguination. The animals were perfused with ice-cold PBS via the aorta, the left kidneys were decapsulated and minced finely with a sterile scissors until tissue was homogenous and clumps were broken down. 65 mg of the minced tissue was placed in 2 ml of ice-cold digestion buffer (10 mg/mL of cold active protease from *Bacillus licheniformis* (Sigma, P5380), 5mM CaCl2 and 125U/ml of DNAse 1 in PBS).[1] The digest mix was incubated on ice and triturated with 1ml pipet tip until the clumps were no longer observed (15 sec every 2 min). Then, the cell suspensions were transferred to 15ml conical tubes and 3ml of ice-cold 10% FBS/PBS was added to inhibit the enzyme activity. The cell suspensions were filtered through 30μM Miltenyi filter rinsed with 4ml of ice-cold

10% FBS/DPBS to ensure the maximal cell yield and pelleted by centrifugation at 300 g for 5 minutes at 4°C. The supernatant was discarded, and the cellular pellet re-suspended in 1ml of ice-cold 0.01% BSA/PBS for Drop-seq and 10% FBS/DPBS for 10x Chromium at 100,000 cells/mL and to 1,000,000 cells/ml, respectively. The remaining minced tissue was snap-frozen in liquid nitrogen for molecular analysis. The contralateral kidneys were fixed with 4% paraformaldehyde (PFA) in PBS overnight (O/N) at 4°C and paraffin embedded for histological assessment. Independent cohorts of identical UIR, UUO and naïve mice (n=4-6 per group) were harvested at the same time point for validation.

scRNA-seq data analysis. The fastq files were processed using 10x Genomics Cell Ranger v6.1.2 (10x Genomics) aligning reads to the mouse genome (mm10) to generate a feature count matrix that included intron counts. Ambient RNA was mediated by using the decontX function within the celda package comparing the filtered cells, cells defined by Cell Ranger, to all other barcoded reads.[2] The resulting cleaned cells were further cleaned by removing cells identified as potential doublets using the doubletFinder package with default settings assuming a 7.5% doublet occurrence per data set.[3] Cell-type clusters and marker genes were identified using the R v4.2.1 library Seurat v4.1.2.[4] Initial cell filtering selected cells that expressed >500 genes. Genes included in the analysis were expressed in a minimum of three cells. Only one read per cell was needed for a gene to be counted as expressed per cell. Cells containing high percentages of mitochondrial, >25%, and hemoglobin genes, >2.5% were filtered out. Each sample was normalized with SCTransform, using the glmGamPoi method regressing out the nCount_RNA variable to minimize the effect of read depth. The final total number of 48590

cells (control: 19025, UIR: 18865, UUO: 10700) were analyzed. Samples were integrated using anchor genes, features common among the samples to preserve cell type identities and to minimize batch effects. All clustering was unsupervised, without driver genes. Genes with the highest variability among cells were used for principal components analysis. Cell clusters were determined by the Louvain algorithm by calculating k-nearest neighbors and constructing a shared nearest neighbor graph, with a resolution set at 0.3. Dimension reduction was performed using UMAP (Uniform Manifold Approximation and Projection) using the first thirty principal components. Marker genes were determined for each cluster using the Wilcoxon Rank Sum test within the FindAllMarkers function using genes expressed in a minimum of 25% of cells and fold change threshold of 1.3. Data are available at Gene Expression Omnibus (GEO) under accession number GSE202882.

Putative signaling interactions between renal cell populations were assessed. Potential receptor-ligand interactions were found by pairing a cell-type expressing a ligand with a cell-type expressing its receptor pair. A receptor or ligand was considered expressed in a cell-type having an average expression of >0.24. Receptor-ligand pairs were determined using the curated receptor-ligand database by the RIKEN FANTOM5 project.[5] Receptor-ligand pairings for each cell type were visualized by a chord diagram using the R package circlize.[6] GO analysis was performed in the ToppGene Suite[7] with 0.05 p value cutoff. The gene clusters enriched in renal cell populations were generated using ToppCluster[8] with Bonferroni correction and 0.05 p value cutoff. The ToppCluster graphs were generated with Fruchterman-Reingold graph layout algorithm, showing individual

genes associated with biological processes enriched in the renal cell population of interest. Venn diagrams were made using InteractiVenn tool.[9]

Renal blood flow procedure (RBF). The independent cohort of UIR, UUO and control mice was subjected to invasive hemodynamics. The mice were anesthetized with ketamine and inaction, femoral artery was cannulated for blood pressure monitoring and femoral vein was used for bolus infusion of medicines, including phenylephrine (PE) used for vasoconstriction, sodium nitroprusside (SNP) used for vasodilation, and dobutamine (DOB) for cardiac stress test. These medicines were administered to examine RBF alterations in the fibrotic and normal kidneys after altering the vascular tone and cardiac activity, as well as to measure basal cardiovascular parameters in the injured and control groups. To access the left kidney, a flank incision was made and the kidney was retracted to expose the renal artery. Then, an ultrasonic flow probe (Transonic systems) was placed around the left renal artery and positioned to obtain maximal blood flow. Mice were given 3-5 min between each dose to ensure the return to the baseline. 4% BSA at 0.15ul/min/gBW was used as maintenance infusion.

Magnetic resonance imaging (MRI). Mice were scanned using a horizontal 7T Biospec MRI system (Bruker, Billerica, MA) with a home-built 35 mm diameter quadrature volume transmit/receive coil. Mice were anesthetized with isoflurane and kept warm with circulating air, which was controlled by a temperature and respiration rate monitor (Small Animal Instruments, Inc., NY). Respiration was maintained around 100 breaths/minute. The mouse kidney was positioned at magnet and coil isocenter. Axial images were

acquired using a fast spin echo sequence with a repetition time of 2500 ms, echo time of 40.2 ms, echo train length of 16, 4 averages, 32 mm x 32 mm field of view, and an acquisition matrix of 200 x 200.

Real-time quantitative PCR (RT-qPCR). Total RNA was isolated from homogenized control, UIR and UUO whole kidney lysates (n=4-6 per group) with RNA Stat-60 extraction reagent (Amsbio, CS-111) and purified using the GeneJET RNA purification kit (ThermoFisher Scientific, KO732). Total RNA and protein were simultaneously isolated from RPTECs using Ambion PARIS kit (AM1921, ThermoFisher Scientific). cDNA was synthesized with the iScript Reverse Transcription Supermix (Bio-Rad, 1708841). qPCR was performed with TaqMan universal PCR master mix (Thermo Fisher Scientific, 4304437) on the Applied Biosystems Quant Studio 3 system. The reported Ct values are the mean of two cDNA sample replicates. The target gene Ct values were normalized to the eukaryotic 18S rRNA endogenous control and presented as the fold change.

Single molecule fluorescent *in situ* hybridization (smFISH), using RNAscope. RNAscope probes and Multiplex Fluorescent v2 assay (323100) were purchased from Advanced Cell Diagnostics, Icn (ACD). Freshly sectioned PFPE 6 µm kidney sections underwent deparaffinization, dehydration, endogenous peroxidase quenching, heat-induced target retrieval and protease digestion, followed by incubation with up to four target riboprobes for 2 hours at 40°C. All the aforementioned steps were performed in RNase-free conditions. Next, tyramide signal amplification and conjugation to an Opal dye (PerkinElmer) were performed according to the manufacturer's protocol. The sections

were treated with DAPI and mounted with Vectashield antifade mounting medium. The controls were treated with negative control riboprobe and signal amplification reagents alongside the experimental sections. Images were obtained on 60x water immersion (WI) objective at Nyquist resolution on the Nikon Ti-E A1R HD confocal with the resonant scanner and processed with NIS-Elements AR 5.2.00 artificial intelligence denoise algorithm (https://www.microscope.healthcare.nikon.com/resources/application-notes/nikon-nis-elements-denoise-ai-software-utilizing-deep-learning-to-denoise-confocal-data). All images within an experimental group were obtained with the same optical configurations.

Western Blotting. Total protein was extracted from homogenized whole kidney lysates and RPTECs using mammalian protein extraction reagent (MPER, ThermoFisher Scientific, 78501) and Ambion PARIS kit, respectively. Extraction buffers were supplemented with protease (ThermoFisher Scientific, 78430) and phosphatase (Sigma-Aldrich, P5726, P0044) inhibitors. 10-15 µg of protein was separated via PAGE, transferred to PVDF membrane, blocked with 5% non-fat milk and incubated with the target recognizing primary antibodies O/N at 4°C. Then, the membranes were washed with Tris buffered saline 0.02% Tween (TBST) and incubated with the secondary HRP-conjugated antibodies for 1 hour at RT. The target protein levels were normalized to the endogenous control detected with goat anti-Gapdh (AF5718, 1:200) or mouse anti-Gapdh (MAB374, 1:5000) antibody. Following primary antibodies were used: rabbit anti-Sox4 (C15310129, 1:1000), rat anti-Cd24 (ab64064, 1:100), goat anti-Cd45 (AF114, 1:2000), mouse anti-αSma (A5228, 1:1000), rabbit anti-Vim (ab45939, 1:1500). The signal was

visualized using SignalFire ECL reagent (Cell Signaling, #6883) using the ChemiDoc imaging system and Bio-Rad's Image Lab Touch Software. The Western blot images were analyzed using ImageJ software; the target protein signal intensity was normalized to the endogenous control protein (Gapdh) signal intensity and shown as representative bands and individual values. The complete Western blot images are provided in the Supplemental figures S12 and S30.

Immunofluorescence (IF). IF staining of fresh PFPE 6 μm kidney sections was performed via deparaffinization, heat induced citrate epitope retrieval, permeabilization in 0.06% Triton-X-100, blocking with 5% serum and O/N incubation with primary antibodies at 4°C. Following primary antibodies were used for IF: rabbit anti-Krt8 (ab53280, 1:100), rabbit anti-Sox4 (ab90696, 1:100), LTA (FL-1321-2, 1:100), DBA (RL-1032-2, 1:100), goat anti-Vcam1 (AF643, 1:50), rat anti-Umod (MAB5175, 1:50), goat anti-Ecad (AF748, 1:20), rabbit anti-Col1a1 (ABT256, 1:100), mouse anti-Myh11 (21404-1-AP, 1:500), rabbit anti-Lrp2 (19700-1-AP, 1:100), mouse anti-Ecad (610182, 1 to 500), rat anti-Lrp2 (31012, 1 to 100). Then sections were incubated with secondary fluorescent antibodies, stained with DAPI, mounted with Vectashield antifade mounting medium and imaged the Nikon Ti-E A1R HD confocal with the resonant scanner. Images were processed with NIS-Elements AR 5.2.00 artificial intelligence denoise algorithm (https://www.microscope.healthcare.nikon.com/resources/application-notes/nikon-nis-elements-denoise-ai-software-utilizing-deep-learning-to-denoise-confocal-data). All images within an experimental group were obtained with the same optical configurations.

Picrosirius Red staining was performed according to the standard protocol and imaged on the Nikon Ti2 wide-field microscope.

Data availability. scRNA-seq data were deposited at the Gene Expression Omnibus under accession number GSE198621.

Statistical Analysis. scRNA-seq was reproduced in three independent runs using DropSeq and 10x Chromium platforms. scRNAseq identified gene expression changes were validated in two separate cohorts of identical UIR, UUO and control mice (n=4-6 per group per cohort). Tubular injury markers Krt8 and Vcam1 were quantified in 3-4 Z-stack high-resolution images per animal, n=4 animals per group. *P* values were generated using Student's t-test with *p<0.05 as statistical significance. Data are shown as individual values, mean ± SD.

Supplementary References

1. Potter AS, Steven Potter S. Dissociation of Tissues for Single-Cell Analysis. *Methods Mol Biol*. 2019;1926:55-62. doi:10.1007/978-1-4939-9021-4_5
2. Yang S, Corbett SE, Koga Y, et al. Decontamination of ambient RNA in single-cell RNA-seq with DecontX. *Genome Biol*. Mar 5 2020;21(1):57. doi:10.1186/s13059-020-1950-6
3. McGinnis CS, Murrow LM, Gartner ZJ. DoubletFinder: Doublet Detection in Single-Cell RNA Sequencing Data Using Artificial Nearest Neighbors. *Cell Syst*. Apr 24 2019;8(4):329-337 e4. doi:10.1016/j.cels.2019.03.003
4. Stuart T, Butler A, Hoffman P, et al. Comprehensive Integration of Single-Cell Data. *Cell*. Jun 13 2019;177(7):1888-1902 e21. doi:10.1016/j.cell.2019.05.031
5. Lizio M, Harshbarger J, Shimoji H, et al. Gateways to the FANTOM5 promoter level mammalian expression atlas. *Genome Biol*. Jan 5 2015;16(1):22. doi:10.1186/s13059-014-0560-6
6. Gu Z, Gu L, Eils R, Schlesner M, Brors B. circlize Implements and enhances circular visualization in R. *Bioinformatics*. Oct 2014;30(19):2811-2. doi:10.1093/bioinformatics/btu393
7. Chen J, Bardes EE, Aronow BJ, Jegga AG. ToppGene Suite for gene list enrichment analysis and candidate gene prioritization. *Nucleic Acids Res*. Jul 2009;37(Web Server issue):W305-11. doi:10.1093/nar/gkp427

8. Kaimal V, Bardes EE, Tabar SC, Jegga AG, Aronow BJ. ToppCluster: a multiple gene list feature analyzer for comparative enrichment clustering and network-based dissection of biological systems. *Nucleic Acids Res*. Jul 2010;38(Web Server issue):W96-102. doi:10.1093/nar/gkq418
9. Heberle H, Meirelles GV, da Silva FR, Telles GP, Minghim R. InteractiVenn: a web-based tool for the analysis of sets through Venn diagrams. *BMC Bioinformatics*. May 22 2015;16(1):169. doi:10.1186/s12859-015-0611-3

Supplemental Tables List and Legends

Table S1. Marker gene lists for all kidney cell populations in the control, UIR and UUO kidneys.

p_val – p value; avg_log2FC - log fold-change of the average expression between the population and all other clusters, positive values indicate that the gene is more highly expressed in the given population; pct.1 - the percentage of cells where the gene is detected in the given population; pct.2 - the percentage of cells where the gene is detected in all other clusters; p_val_adj - adjusted p-value, based on Bonferroni correction using all genes in the dataset; cluster – identified cluster name; gene – gene name.

Table S2. Ligand-receptor interactions in the control, UIR and UUO kidneys.

lclust - kidney cell population predicted to express gene encoding ligand, rclust - kidney cell population predicted to express gene encoding receptor, lexp - p-Value of ligand encoding gene expression, rexp - p-Value of receptor encoding gene expression.

Table S3. Quantitative analysis of epithelial-to-stromal interactions in the control, UIR and UUO kidneys.

lclust - kidney cell population predicted to express gene encoding ligand, rclust - kidney cell population predicted to express gene encoding receptor. Number of interactions between each epithelial cluster and fibroblast population is listed on the right.

Table S4. Comparative analysis of marker genes expressed in three fibroblast

populations.

Lists of unique and shared fibroblast marker genes displayed in the Venn diagram, along with GO analyses of biological processes enriched among the unique and shared genes.

Table S5. Gene ontology (GO) biological process ToppGene analysis of failed repair tubular epithelial cells (frTECs) marker genes (pleases see Supplementary table S1 for the gene list).

Table S6. Marker gene lists for all kidney cell populations in the WT E18 kidney (GSE214024).

p_val – p value; avg_log2FC - log fold-change of the average expression between the population and all other clusters, positive values indicate that the gene is more highly expressed in the given population; pct.1 - the percentage of cells where the gene is detected in the given population; pct.2 - the percentage of cells where the gene is detected in all other clusters; p_val_adj - adjusted p-value, based on Bonferroni correction using all genes in the dataset; cluster number – number of the cluster on the UMAP; gene – gene name.

Table S7. Comparison of adult frTEC marker genes vs adult proximal tubules or adult distal nephron tubular clusters (loop of Henle, distal tubule, collecting duct principal and intercalated).

Complete lists of genes unique or overlapping between frTECs and other adult tubular epithelial populations shown in Venn diagrams.

Supplemental Figures and Legends

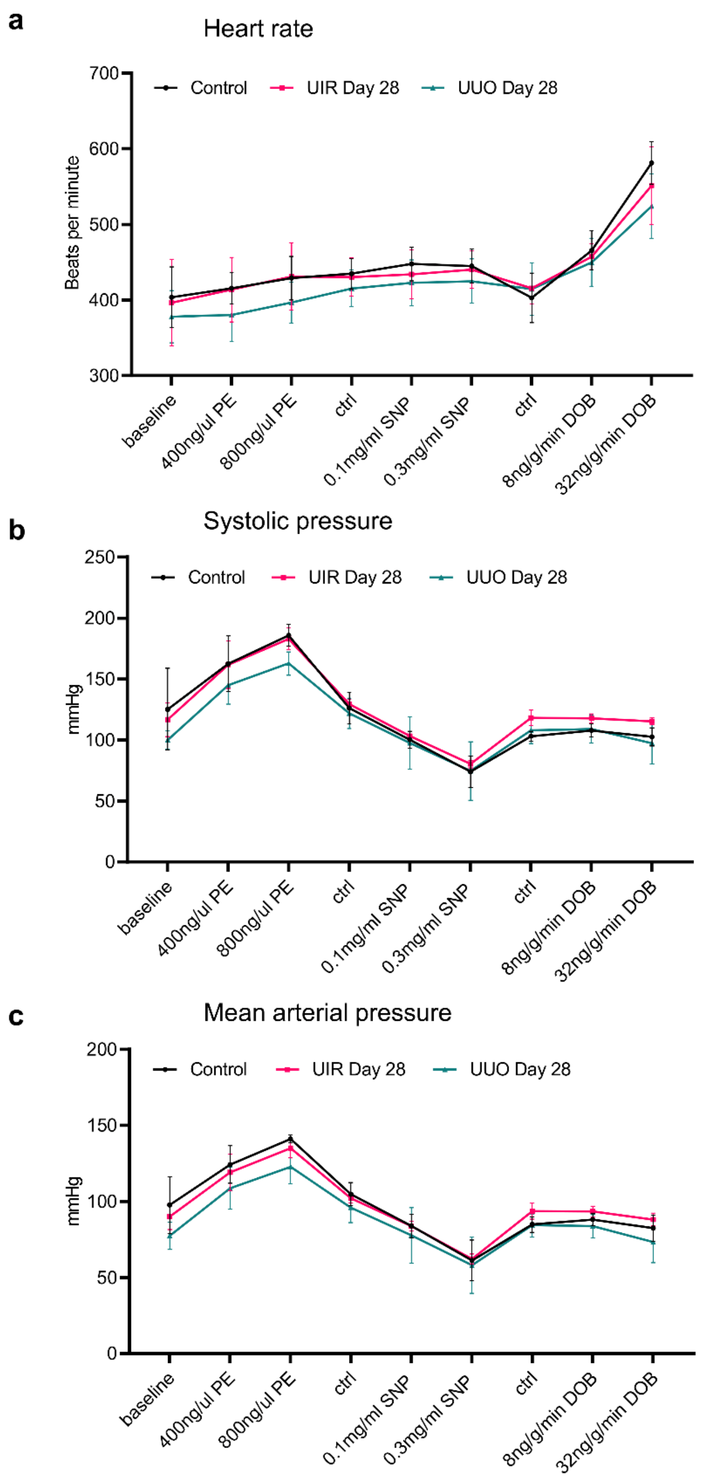

Figure S1. Invasive hemodynamic reveals no significant systemic cardiovascular function changes caused by renal fibrosis models compared to the control. (a) Heart rate in beats per minute (bpm) at baseline, with vasoconstrictive (PE, phenylephrine), vasodilative (SNP, sodium nitroprusside) and inotropic agent (DOB, dobutamine). Ctrl – control interval between agents. Data are presented as mean values ± SD, n=3-4 per group. (b) Mean arterial pressure (mmHg) at baseline, with vasoconstrictive (PE, phenylephrine), vasodilative (SNP, sodium nitroprusside) and inotropic agent (DOB, dobutamine). Ctrl – control interval between agents. Data are presented as mean values ± SD, n=3-4 per group. (c) Systolic pressure (mmHg) at baseline, with vasoconstrictive (PE, phenylephrine), vasodilative (SNP, sodium nitroprusside) and inotropic agent (DOB, dobutamine). Ctrl – control interval between agents. Data are presented as mean values ± SD, n=3-4 per group.

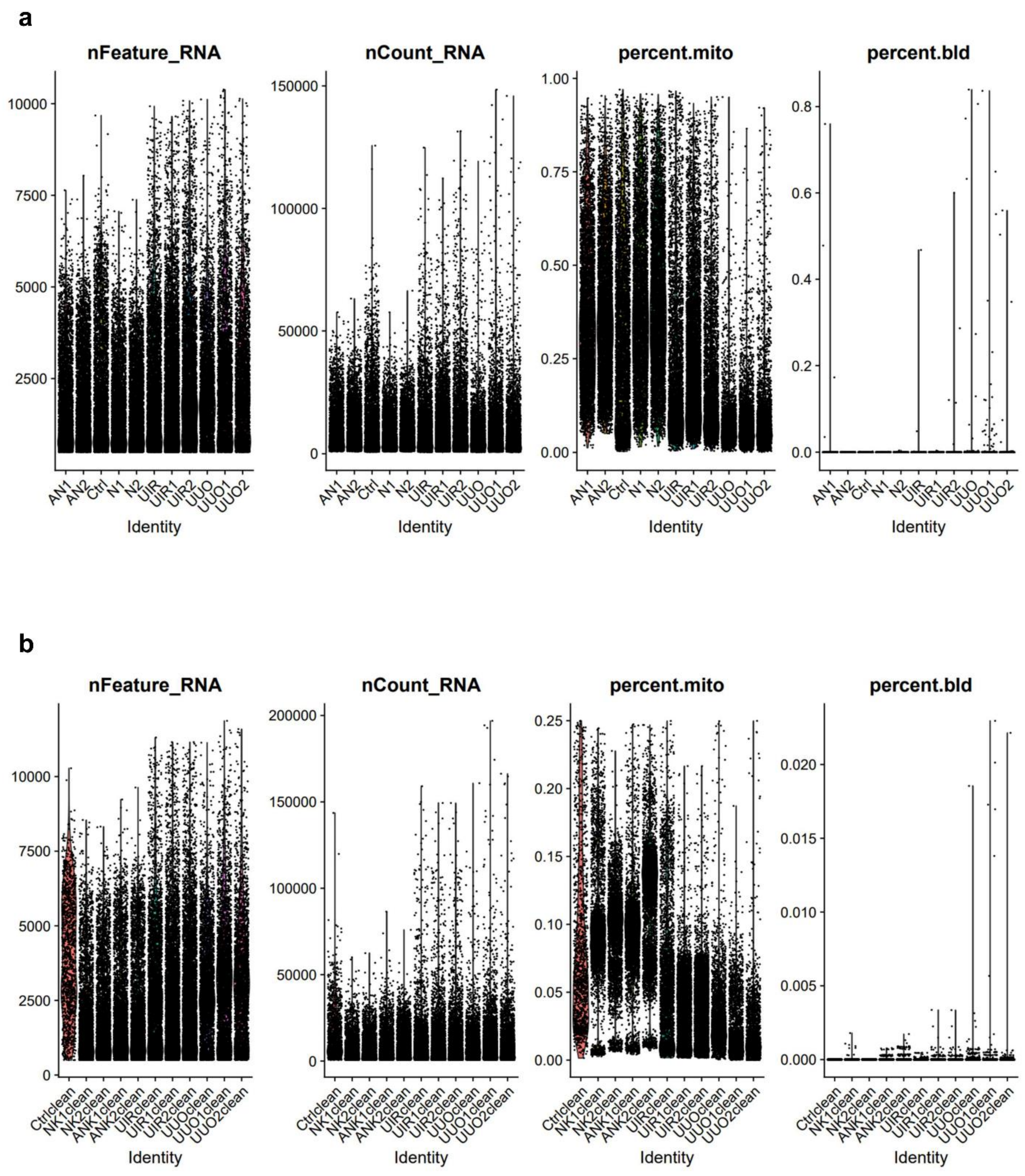

Figure S2. Quality control cutoffs used on scRNA-seq data processing. Cell statistics before (a) and after (b) applying quality control cutoffs. QC_prefiltering contains all cells with a minimum of 500 genes per cell from each sample. All_batch_postcutoff_vlnplot shows the quality metrics of the cells from each sample after background removal, doublet removal, and removing cells with high percentages of mitochondrial genes and hemoglobin genes. Cells containing high percentages of mitochondrial, >25%, and hemoglobin genes, >2.5% were filtered out.

a

b

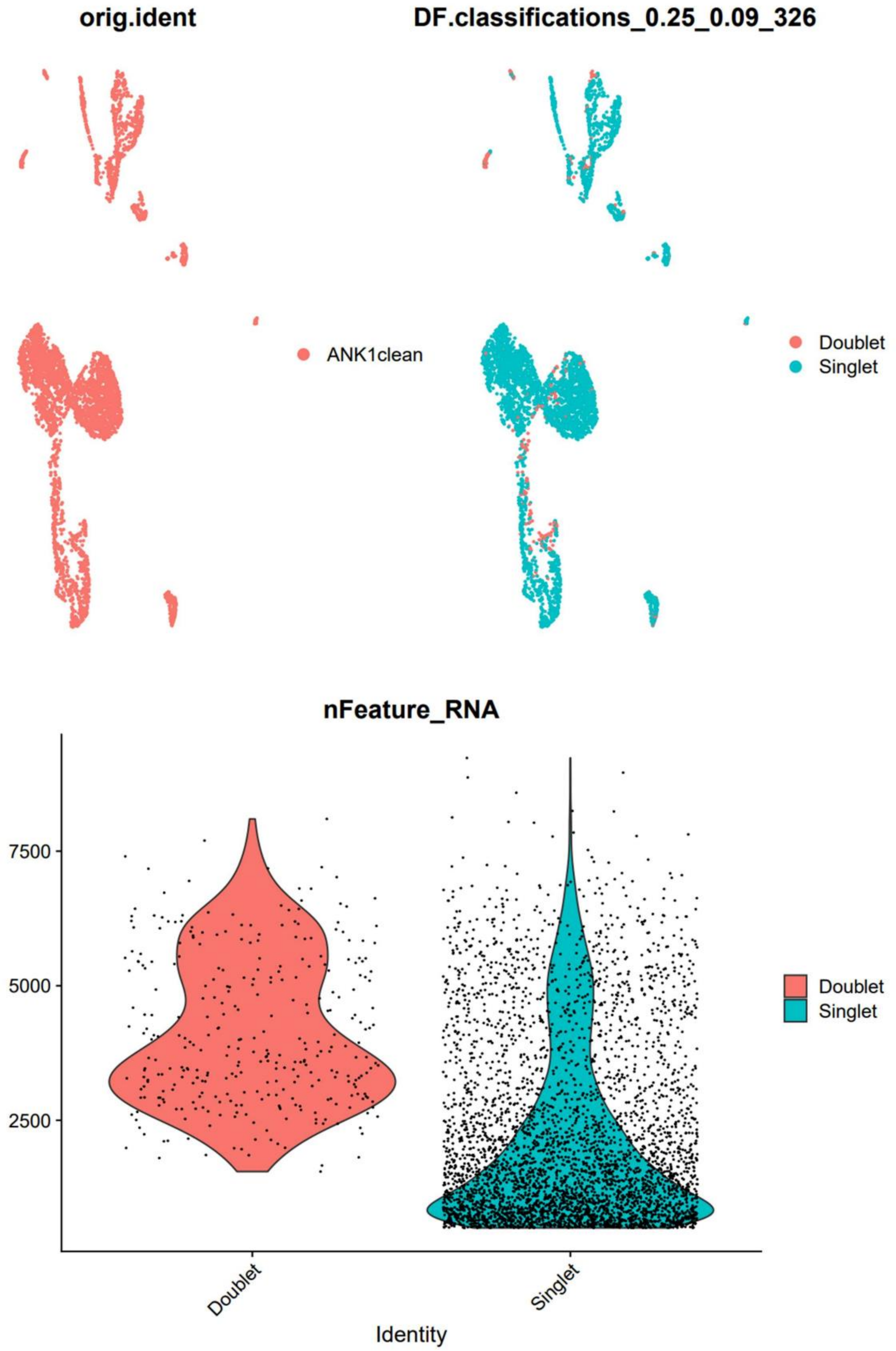

Figure S3. DoubletFinder package is used to identify the potential doublets in scRNA-seq data. (a) ANK1_doubletplot shows the original UMAP of all cells on the left and highlights the cells called doublets by DoubletFinder on the right (salmon color). (b) ANK1_doublets_by_features highlights the number of features found in the doublets vs singlets.

a

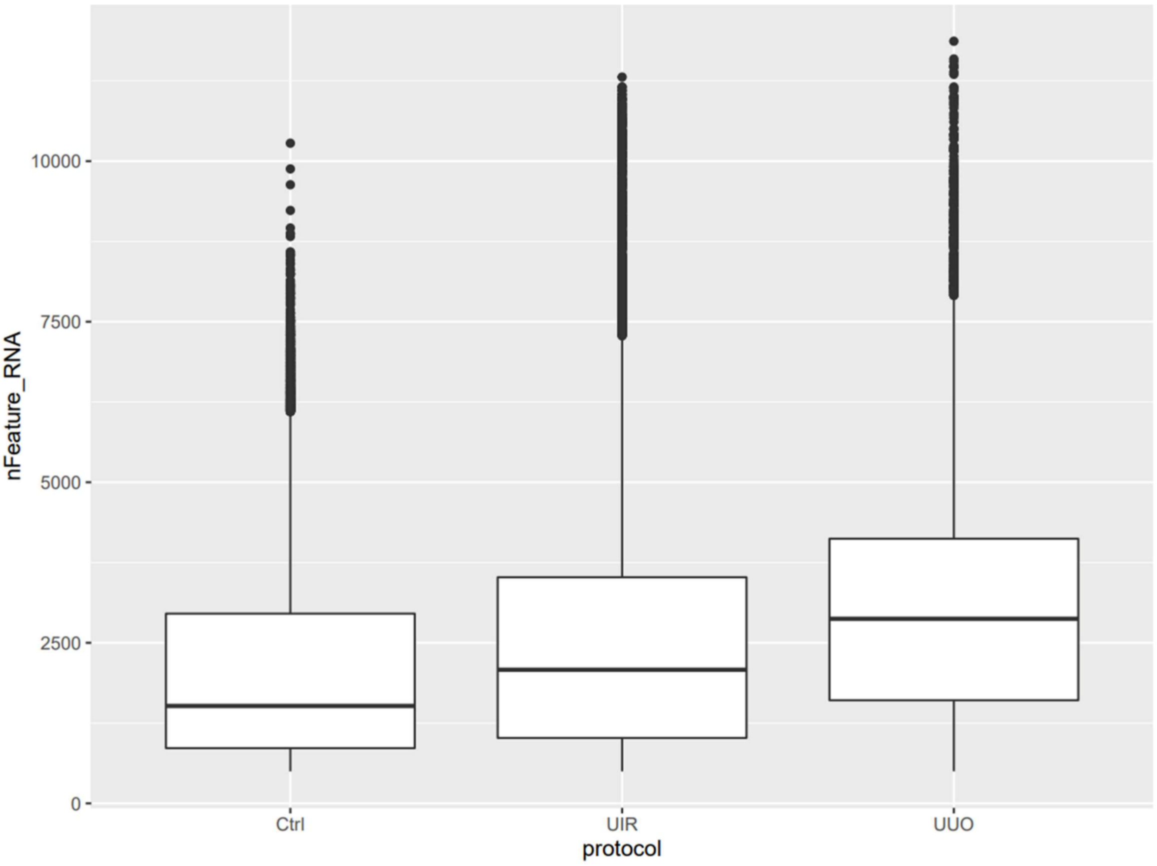

Figure S4. Gene distribution per condition in the control, UIR and UUO scRNA-seq data. (a) Boxplots show the distribution of the number of features (genes) in each condition.

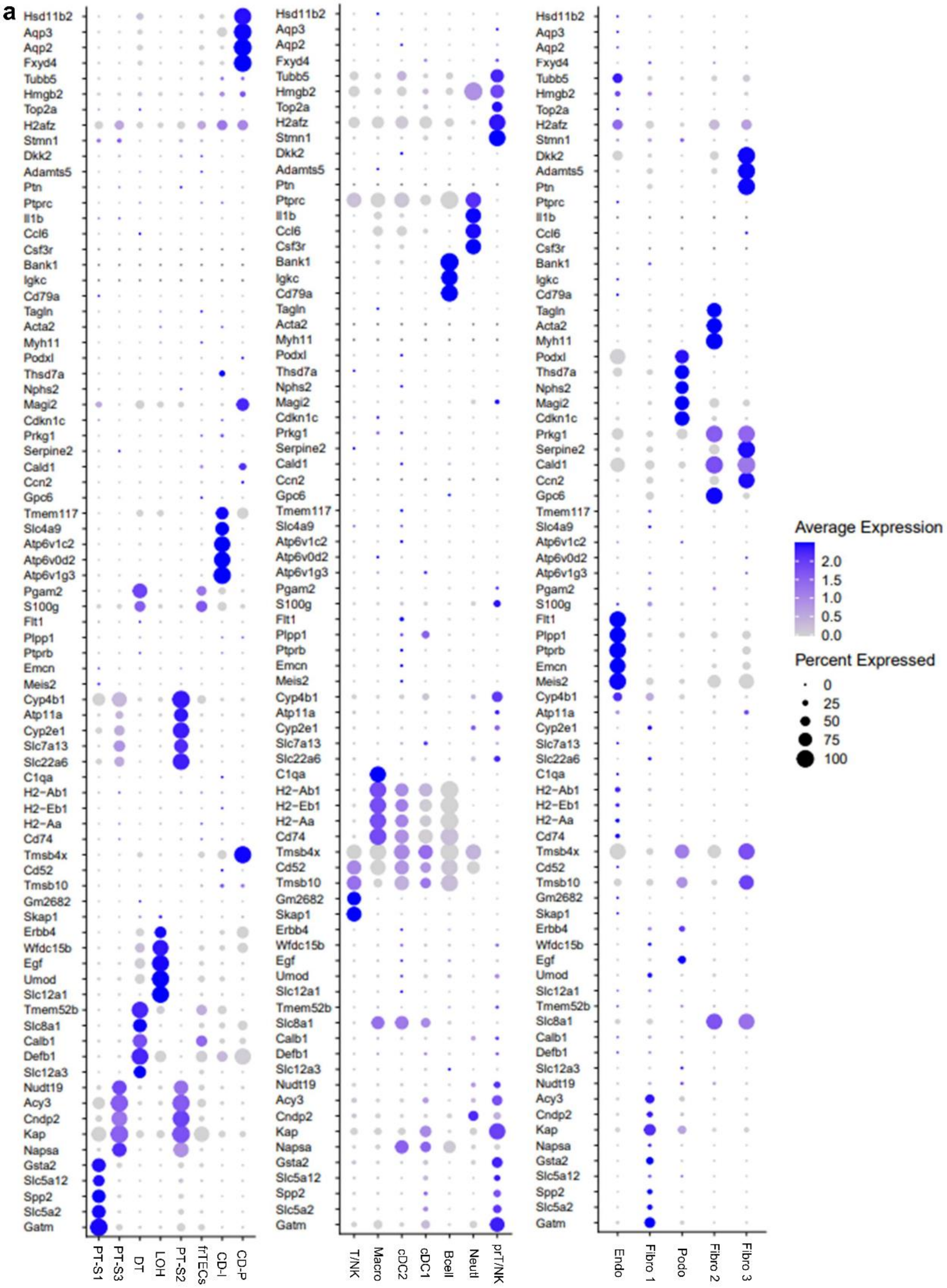

Figure S5. scRNA-seq identifies cellular landscape of the control kidneys. (a) Dot plot of cell type-specific expression of marker genes for manually annotated clusters in the control kidneys. Dot size denotes percentage of cells expressing the marker. Color intensity represents average gene expression values.

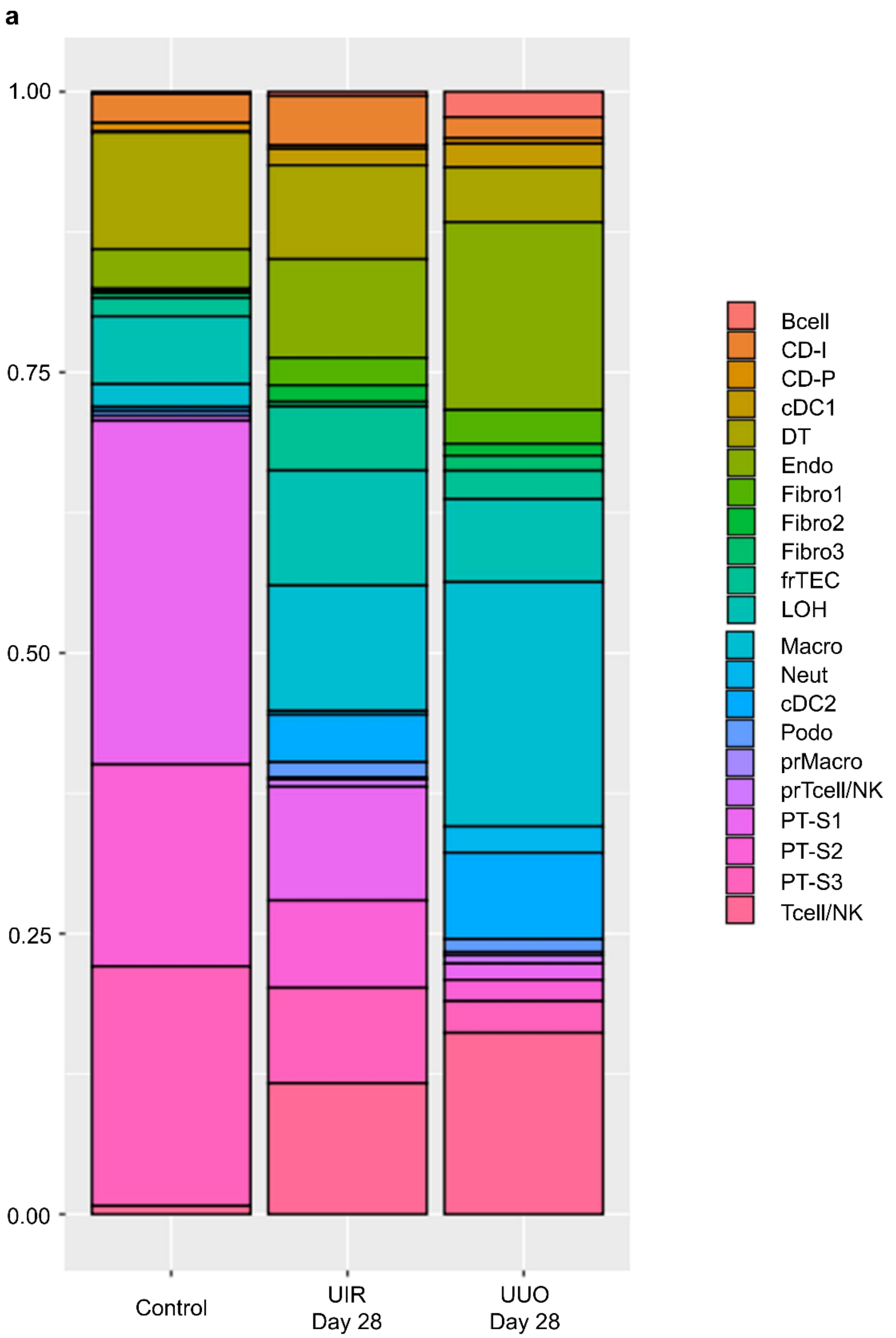

Figure S6. scRNA-seq identifies the fractional composition of cellular clusters in the control, UIR and UUO Day 28 kidneys. (a) Bar plots displaying the proportional abundance of the cell populations in the control, UIR and UUO Day 28 conditions. Cluster names are presented on the right.

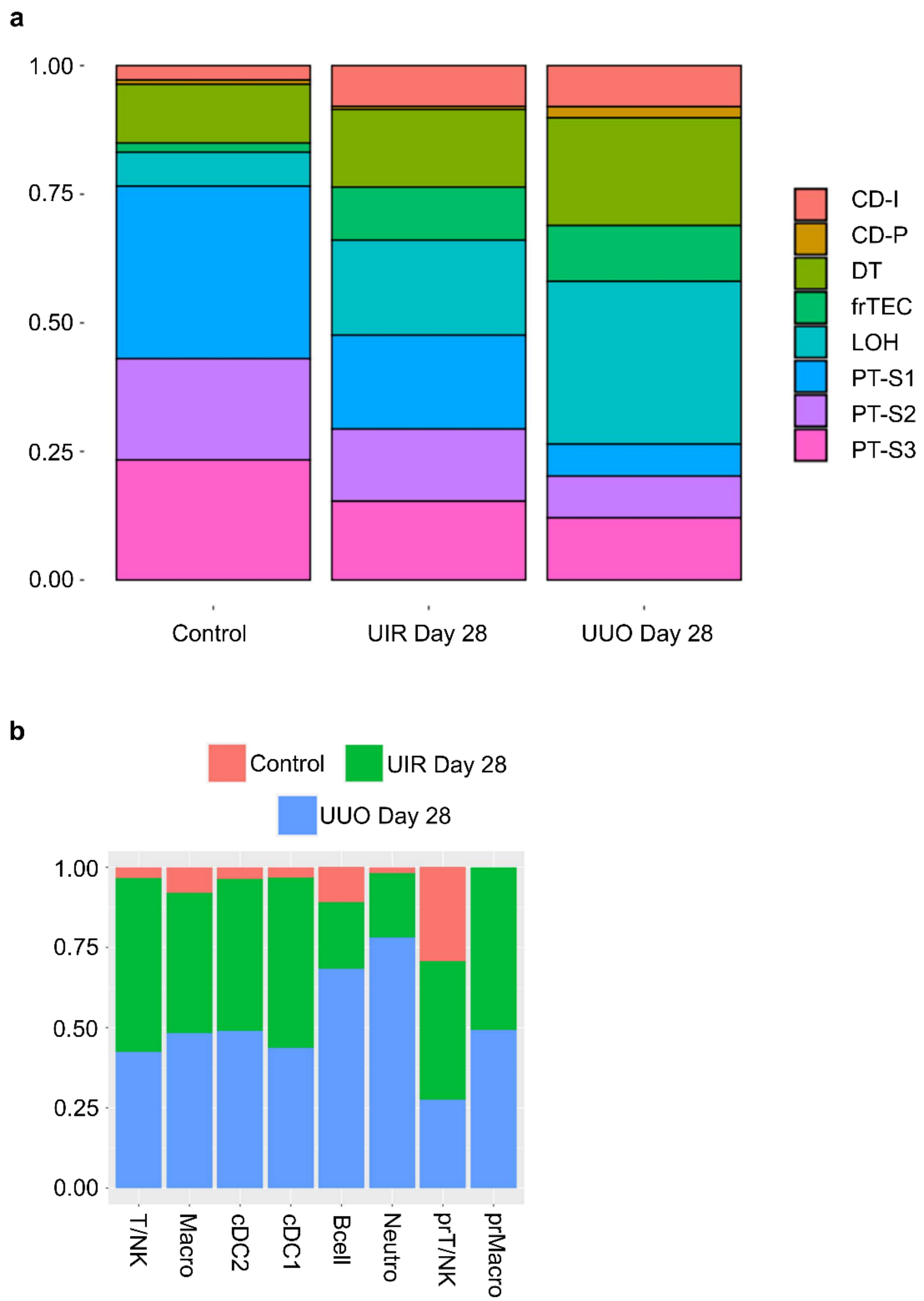

Figure S7. scRNA-seq dissects relative abundance of tubular epithelial populations in the control and fibrotic kidneys. (a) Bar plots displaying the proportional abundance of tubular epithelial populations in the control, UIR and UUO Day 28 conditions. Cluster names are presented on the right. Cell fractional abundances are shown within each condition. (b) Relative immune cluster proportion in the control (salmon), UIR (green) and UUO (blue) kidneys. Cell subset proportion change is shown relative to the listed conditions.

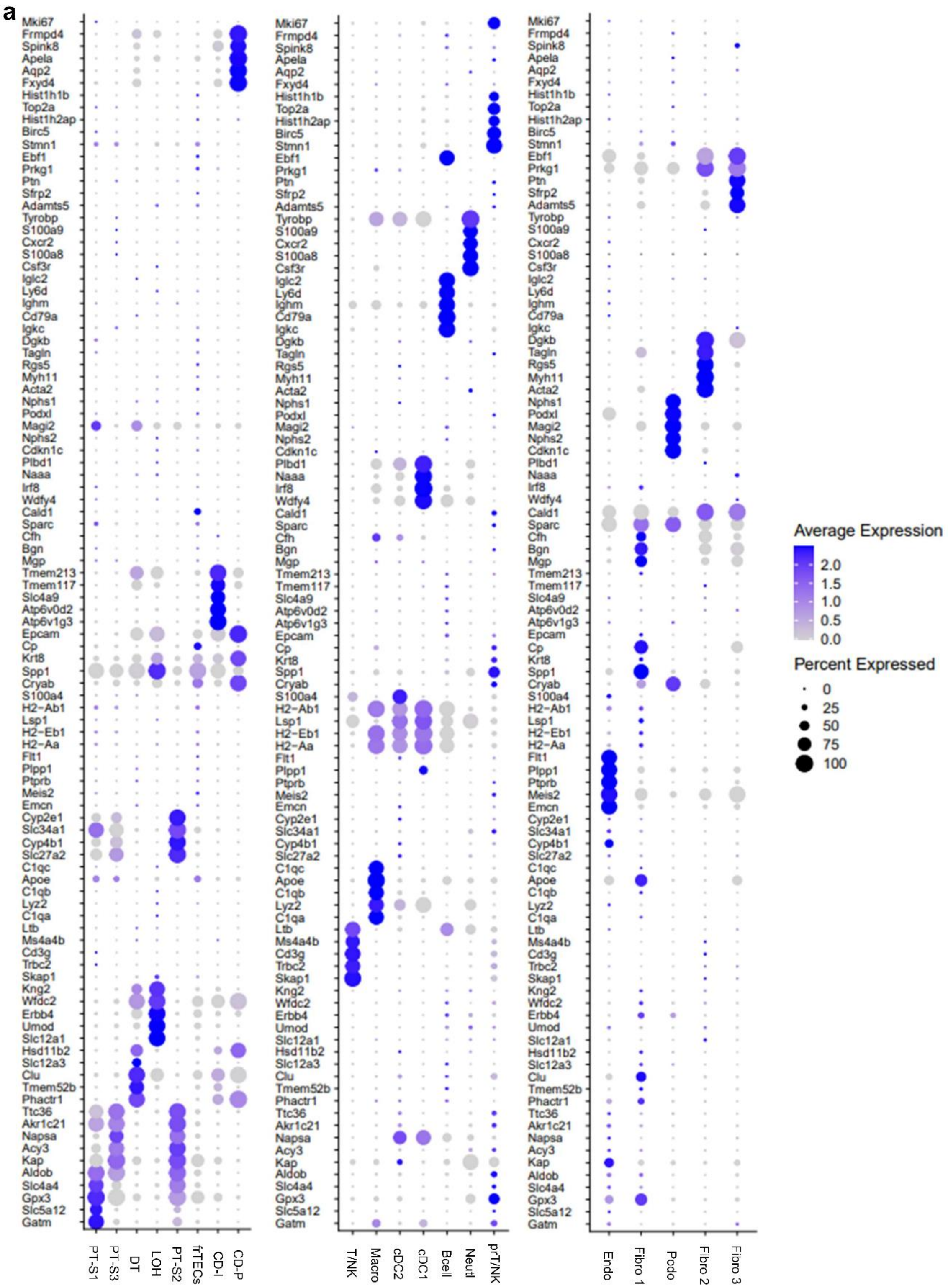

Figure S8. scRNA-seq identifies cellular landscape of the UIR kidneys. (a) Dot plot of cell type-specific expression of marker genes for manually annotated clusters in the UIR kidneys. Dot size denotes percentage of cells expressing the marker. Color intensity represents average gene expression values.

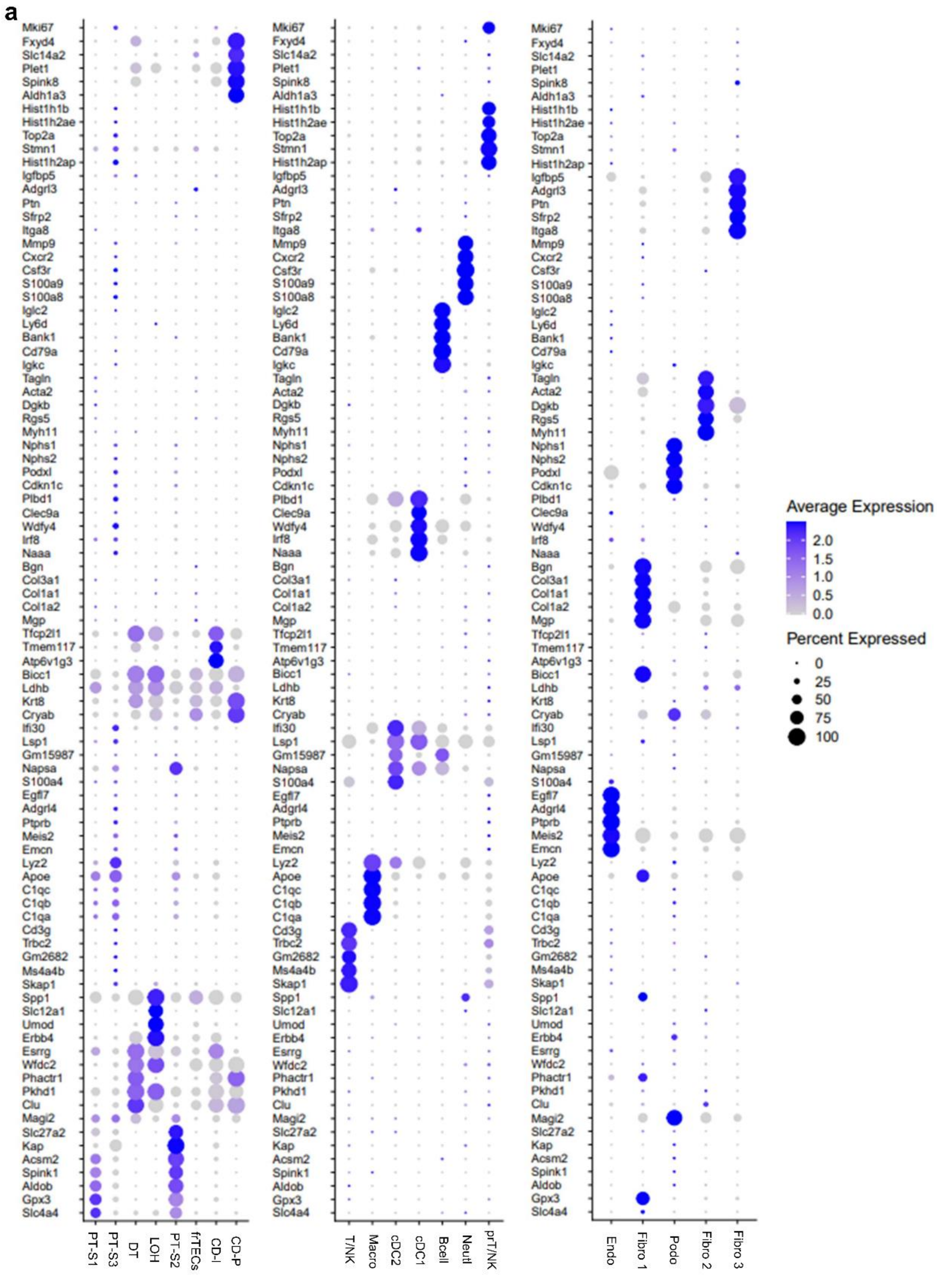

Figure S9. scRNA-seq identifies cellular landscape of the UUO kidneys. (a) Dot plot of cell type-specific expression of marker genes for manually annotated clusters in the UUO kidneys. Dot size denotes percentage of cells expressing the marker. Color intensity represents average gene expression values.

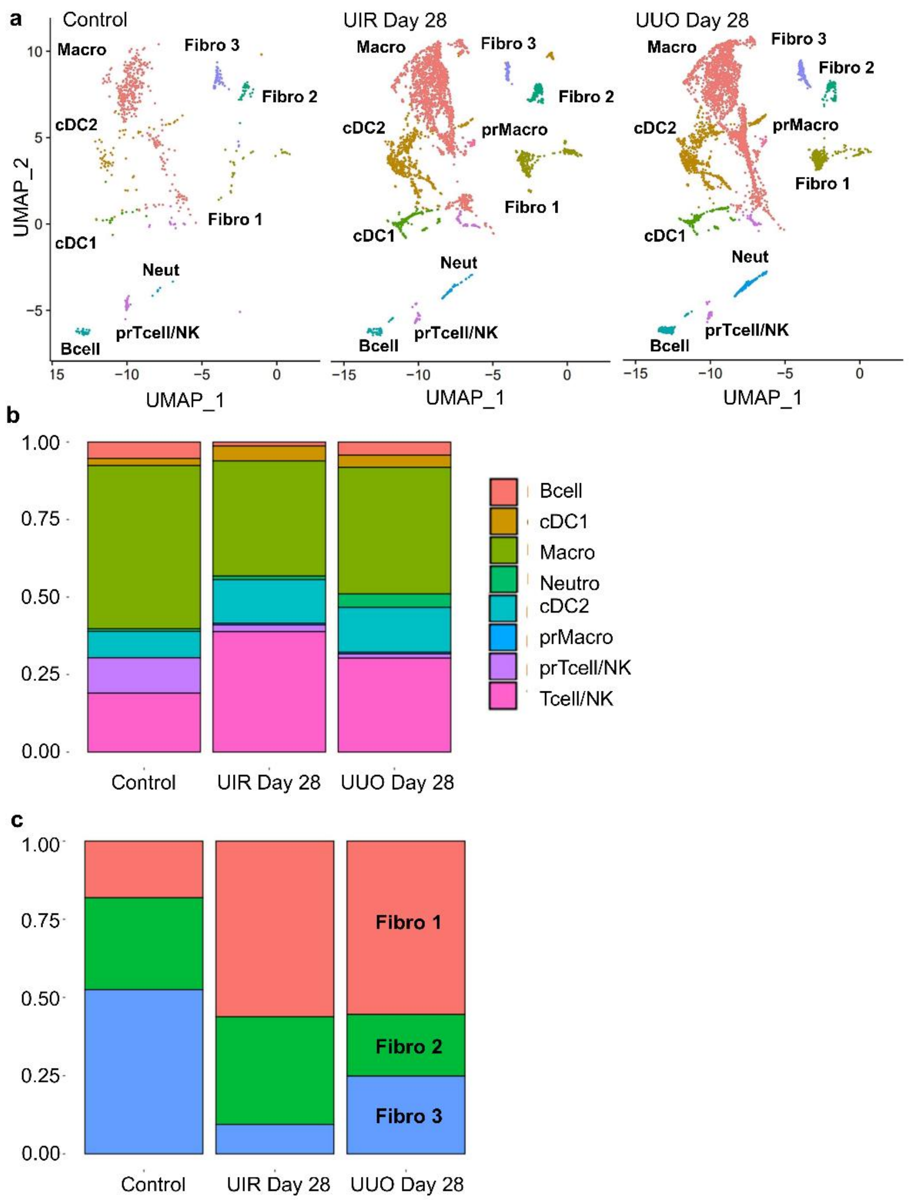

Figure S10. scRNA-seq dissects relative abundance of immune and fibroblast populations in the control and fibrotic kidneys. (a) UMAPs show renal immune and fibroblast cell populations in the control, UIR and UUO kidneys (n=3-5 per group). Clusters are distinguished by different colors. Macro, macrophages, Neutro, neutrophils, cDC, conventional dendritic cells, NK, natural killer, prTcell, proliferating Tcell, Fibro, fibroblasts, prMacro, proliferating macrophages. (b) Bar plots displaying the proportional abundance of the immune populations in the control, UIR and UUO Day 28 conditions. Cluster names are presented on the right. Cell fractional abundances are shown within each condition. Cellular fraction comparison between the control, UIR and UUO kidneys is shown in the figure 2b-d. (c) Bar plots displaying the proportional abundance of the fibroblast populations in the control, UIR and UUO Day 28 conditions. Cluster names are presented on the right. Cell fractional abundances are shown within each condition.

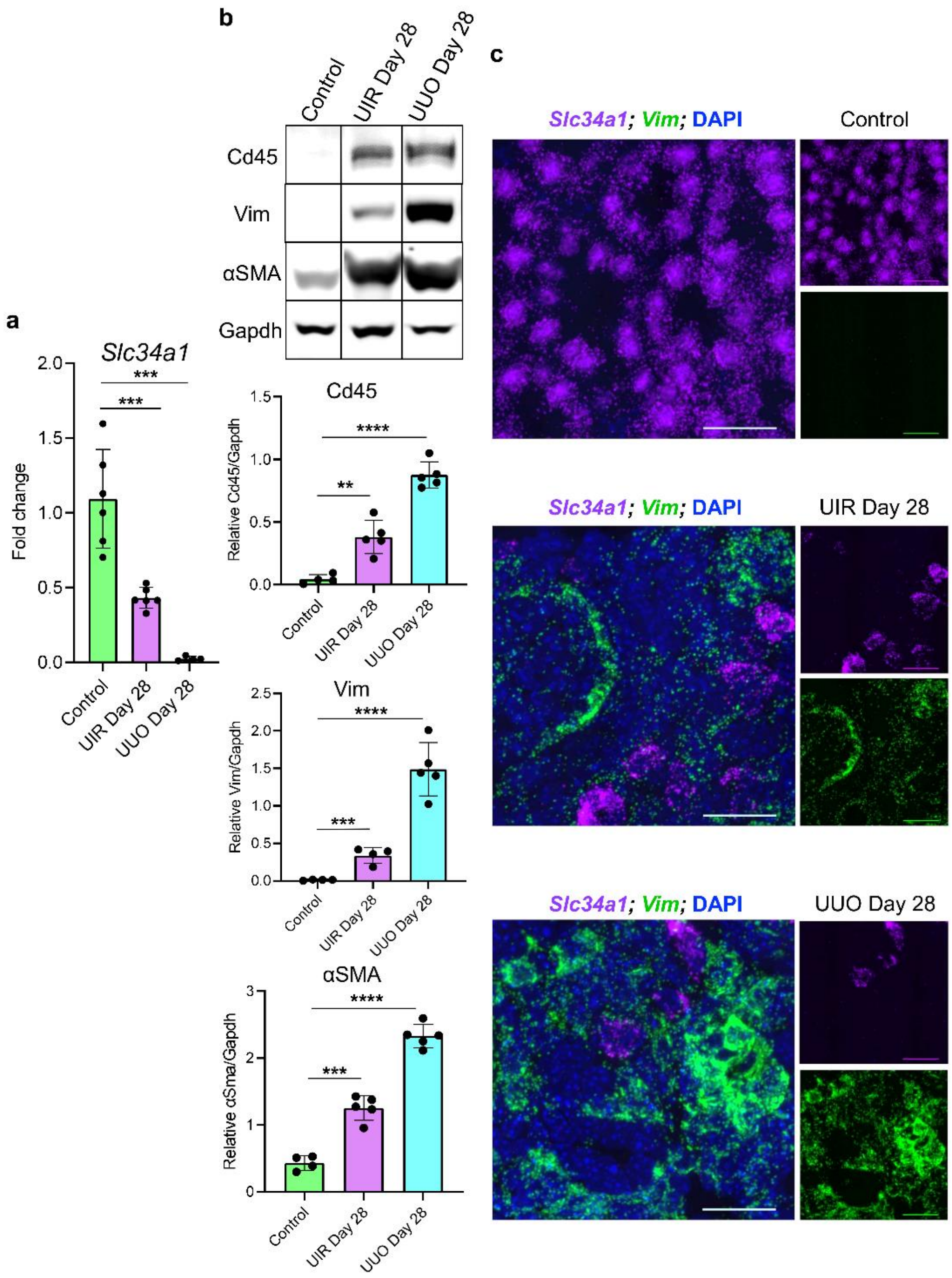

Figure S11. Validation corroborates UIR and UUO induced proximal tubule loss, fibroblast activation and inflammatory infiltration demonstrated by scRNA-seq.
(a) qPCR of the proximal tubule (*Slc34a1*) marker, n=4-7 per group. (b) Representative images and quantifications of Western blots for fibrosis (Vim), myofibroblasts (αSma) and inflammation (Cd45) markers, n=4-5 per group. Representative bands are cropped out of the original gels and are separated by the black border, the unprocessed original blots/gels are presented in Supplementary figure S12. (c) Representative images of *Slc34a1* (purple), *Vim* (green) and DAPI (blue) RNAscope. Original magnification, ×60, 0.07 μm/px Nyquist zoom, maximal intensity projection from approximately 6-μm Z-stacks. $^{**}P \leq 0.01$, $^{***}P \leq 0.001$, $^{****}P \leq 0.0001$ compared to control, Student's t test for (a) and (b).

a

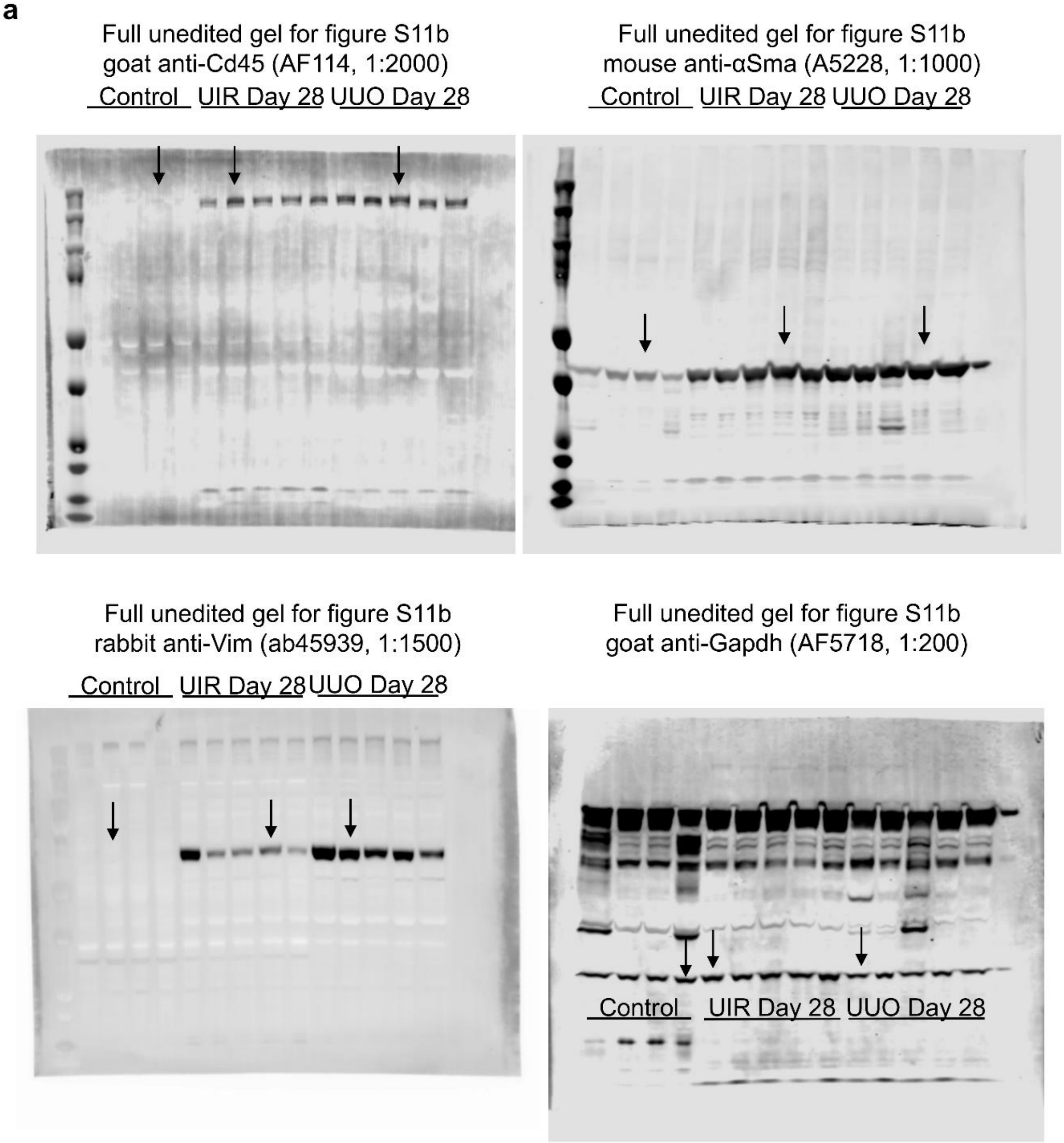

Figure S12. Original uncropped blots for Figure S11b. (a) Original uncropped images of Western blots for fibrosis (Vim), myofibroblasts (αSma) and inflammation (Cd45) markers along with the positive control (Gapdh), n=4-5 per group. Representative bands shown in figure S11b are highlighted with black arrows.

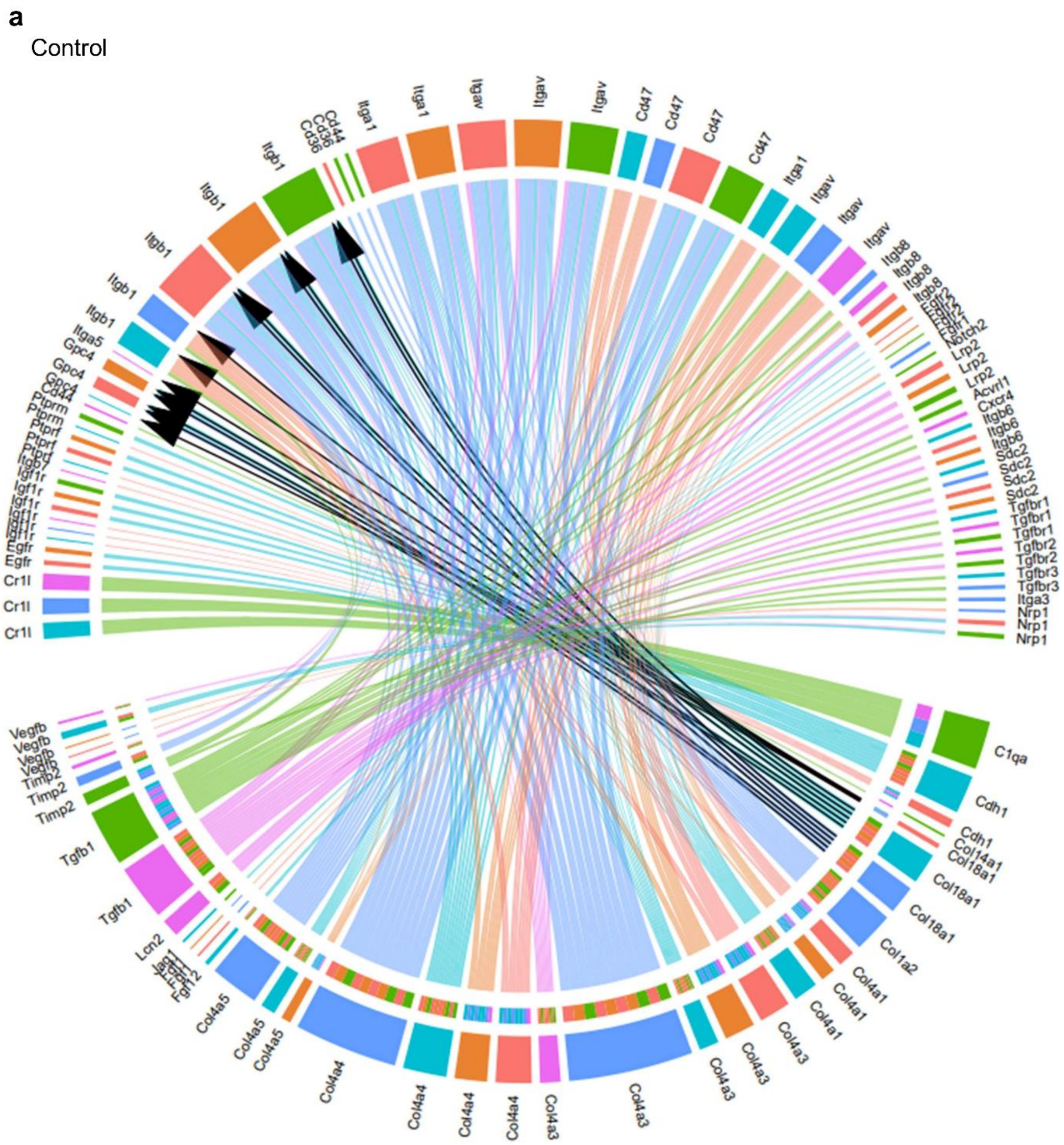

Figures S13-15. scRNA-seq dissects the molecular and cellular nature of the cell-to-cell crosstalk in the normal kidney. Circus plots display proximal-to-stromal **(S11a, current figure)**, distal nephron tubule segment to stromal (S12a) and stromal-to-stromal (S13a) ligand-receptor interactions in the control kidneys. Outer portion of the lower part of the circus plot represents populations expressing ligand encoding gene. Inner portion of the lower part of the circus plot as well as the upper part represents those populations expressing receptor encoding genes. Populations are color-coded identically to the UMAPs in Figure 1c. Black arrows highlight *Col18a1* and its receptor encoding genes.

a

Control

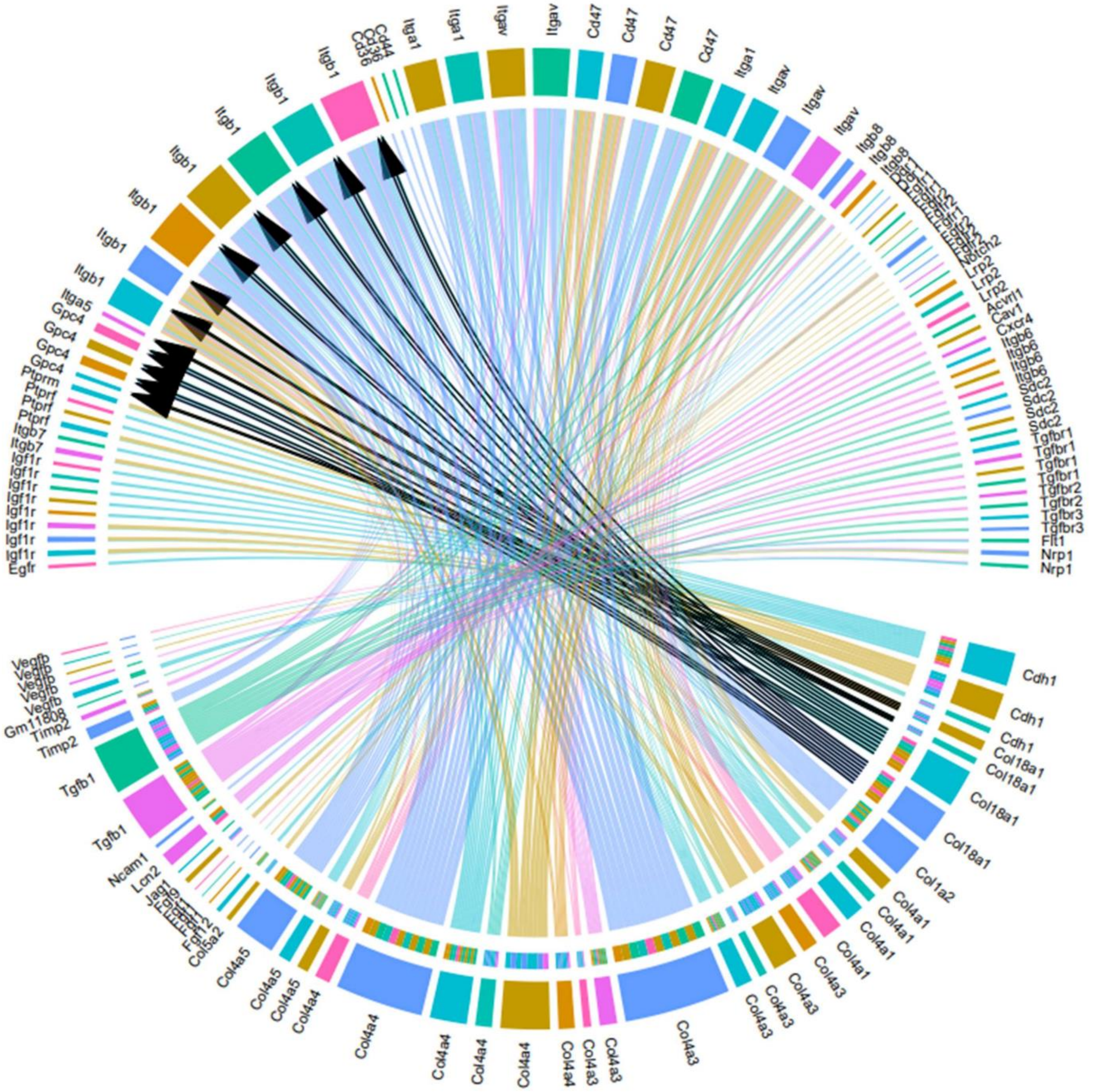

Figure S14. scRNA-seq dissects the molecular and cellular nature of distal nephron tubule segment to stromal cell-to-cell crosstalk in the normal kidney.

a

Control

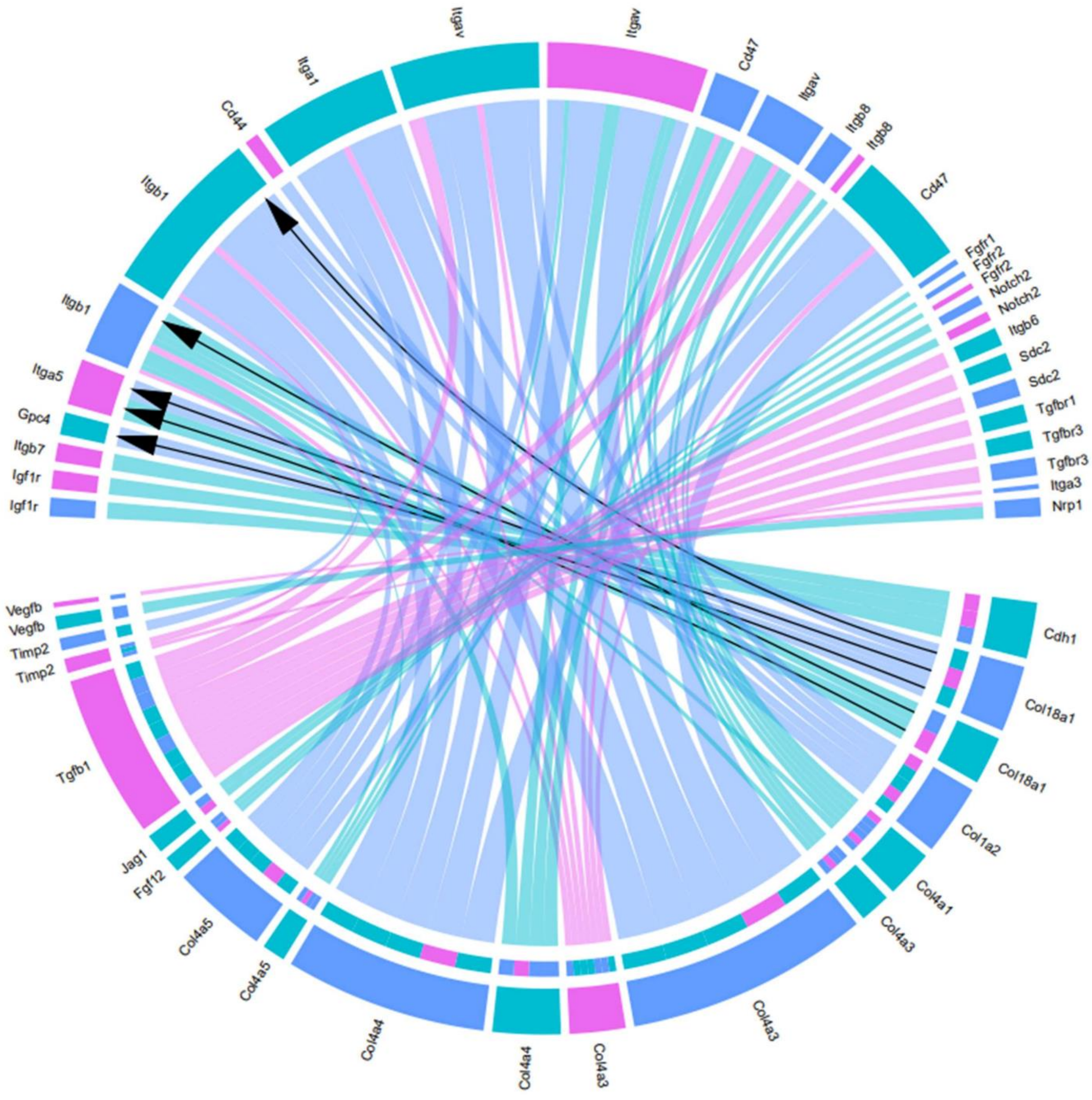

Figure S15. scRNA-seq dissects the molecular and cellular nature of the stromal-to-stromal cell-to-cell crosstalk in the normal kidney.

Figures S16-19. scRNA-seq dissects the molecular and cellular nature of epithelial-to-stromal crosstalk in the fibrotic kidney. Circus plots display proximal-to-stromal (S14a and S15a) and distal nephron tubule segment to stromal (S16a and S17a) ligand-receptor interactions in the UIR and UUO kidneys. Outer portion of the lower part of the circus plot represents populations expressing ligand encoding gene. Inner portion of the lower part of the circus plot as well as the upper part represents those populations expressing receptor encoding genes. Populations are color-coded identically to the UMAPs in Figure 1c. Black arrows highlight *Col18a1* and its receptor encoding genes. Complete lists of scRNA-seq predicted ligand-receptor interactions are presented in the Supplementary table S5. Note the remarkable increase in distal nephron tubule segment to stromal communications in both fibrotic injuries.

a

UIR Day 28

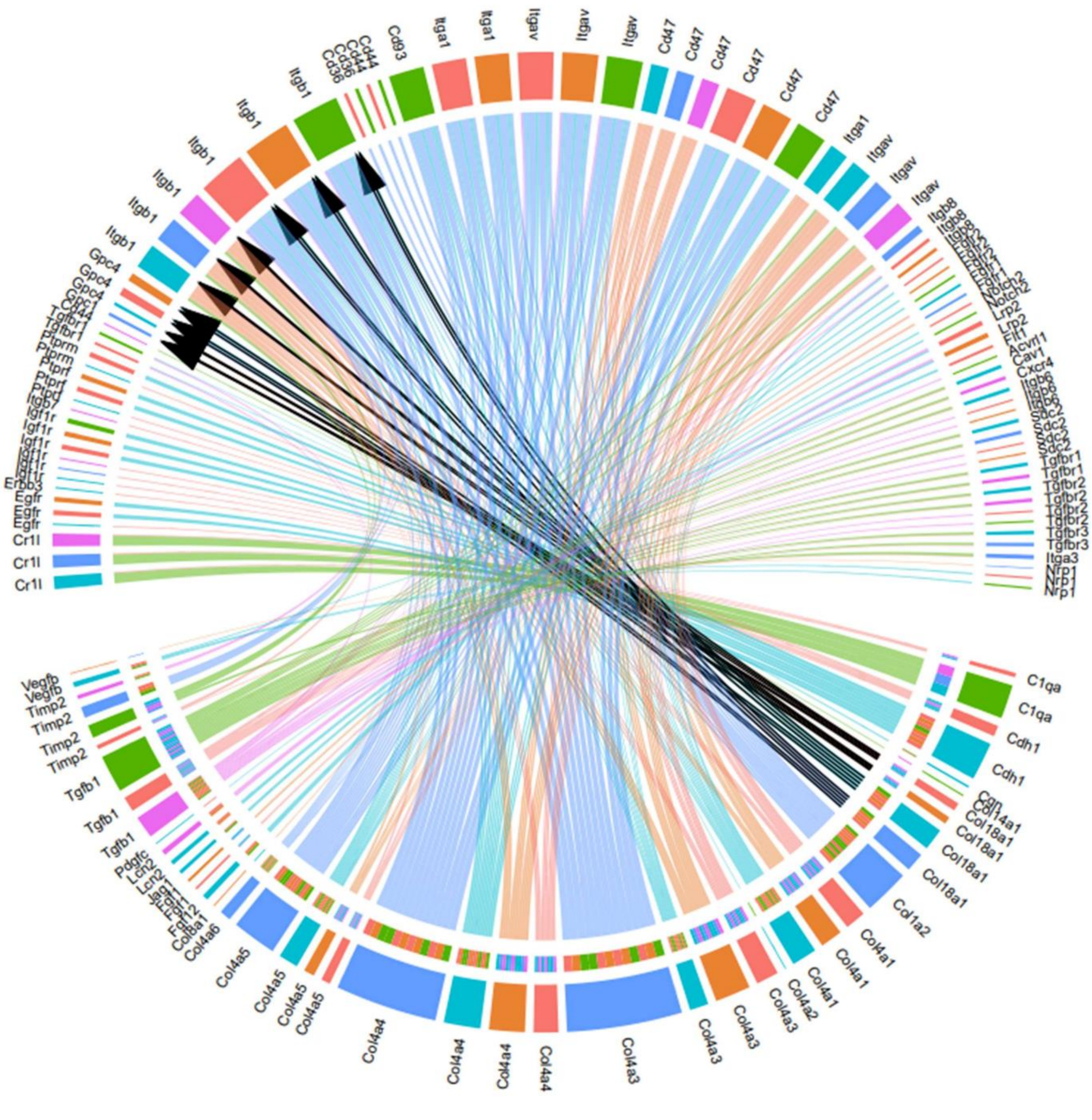

Figure S16. scRNA-seq dissects the molecular and cellular nature of proximal-to-stromal crosstalk in the fibrotic kidney.

a

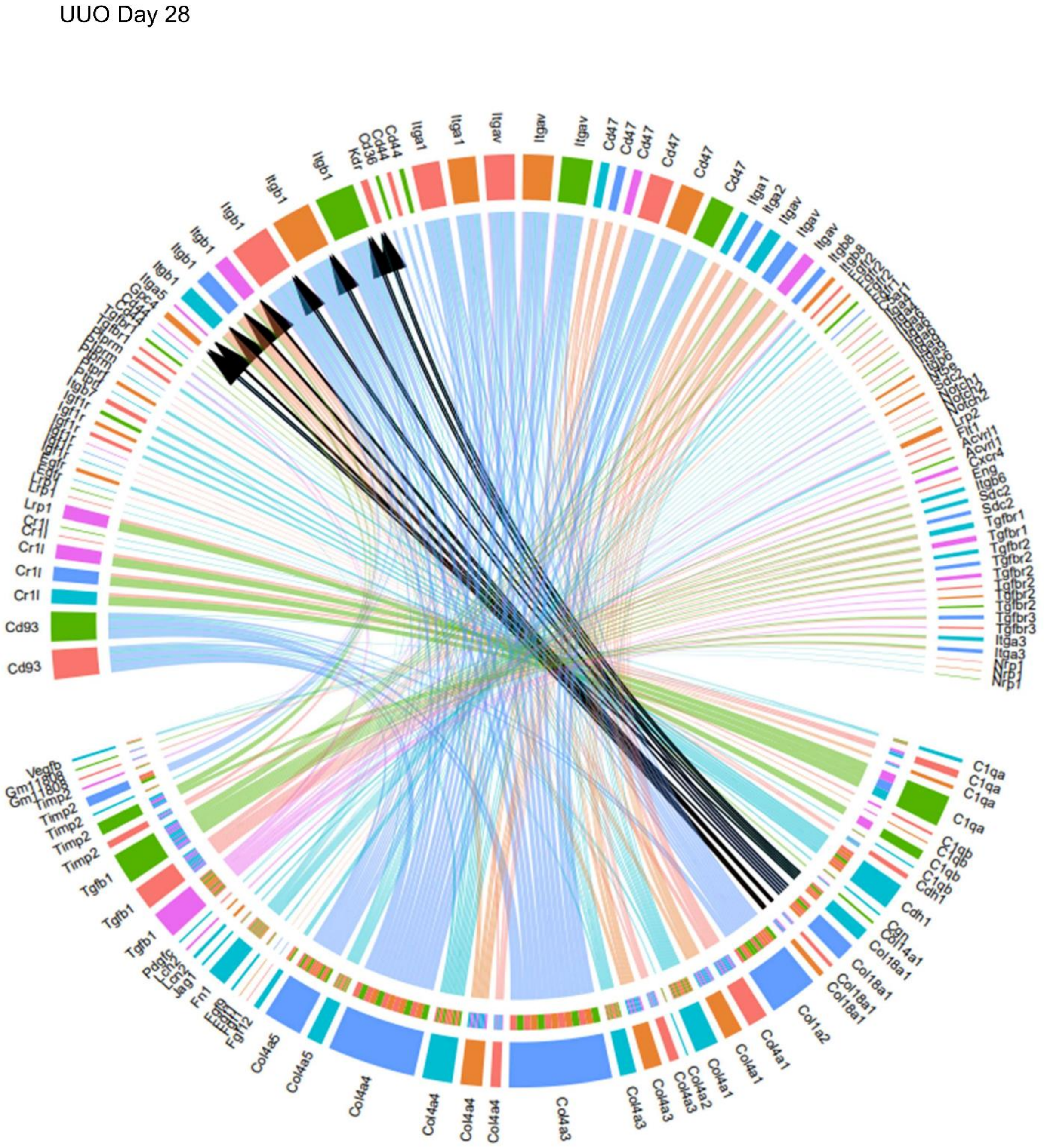

Figure S17. scRNA-seq dissects the molecular and cellular nature of proximal-to-stromal crosstalk in the fibrotic kidney.

a

UIR Day 28

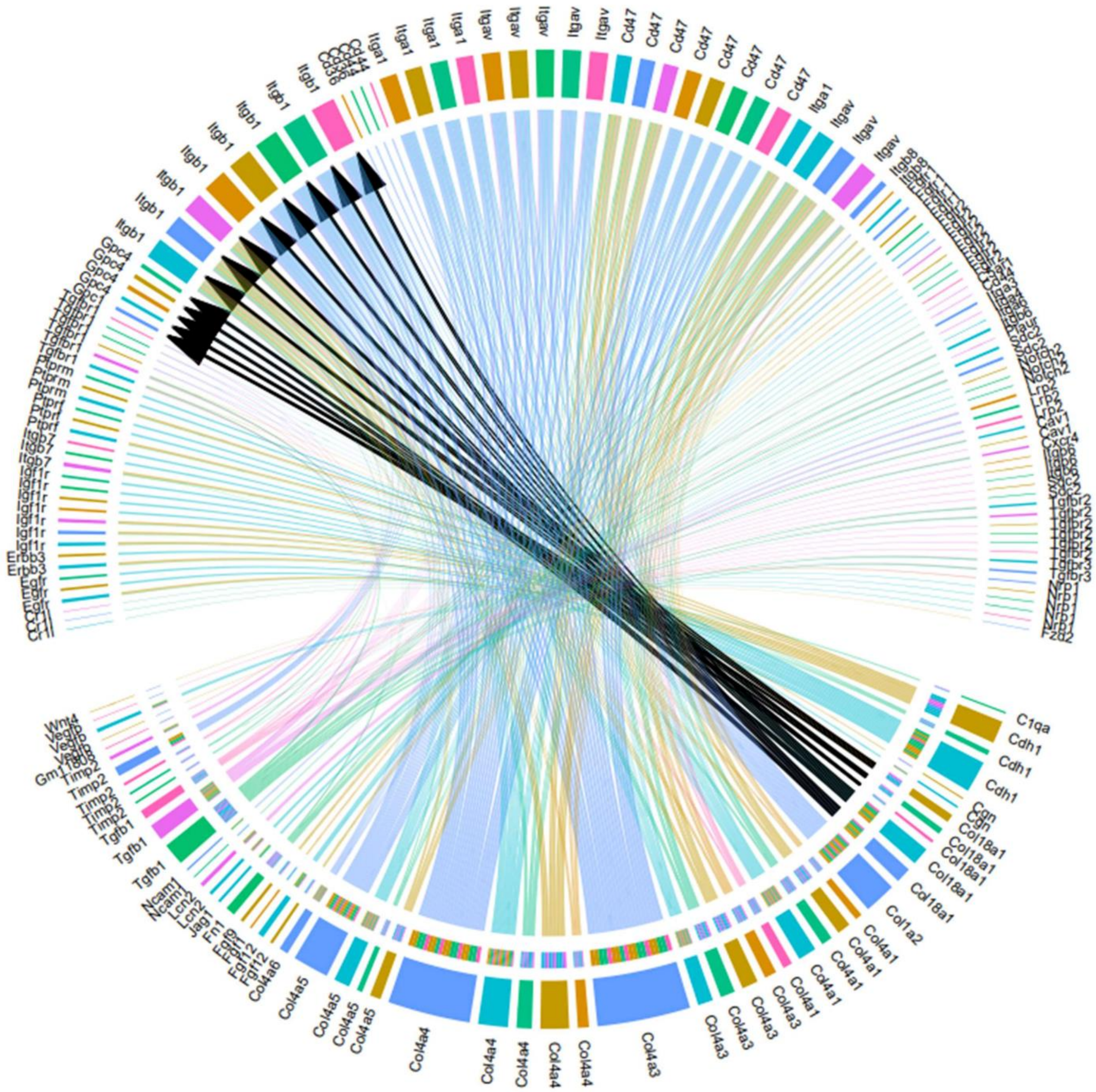

Figure S18. scRNA-seq dissects the molecular and cellular nature of distal nephron tubule segment to stromal crosstalk in the fibrotic kidney.

a

UUO Day 28

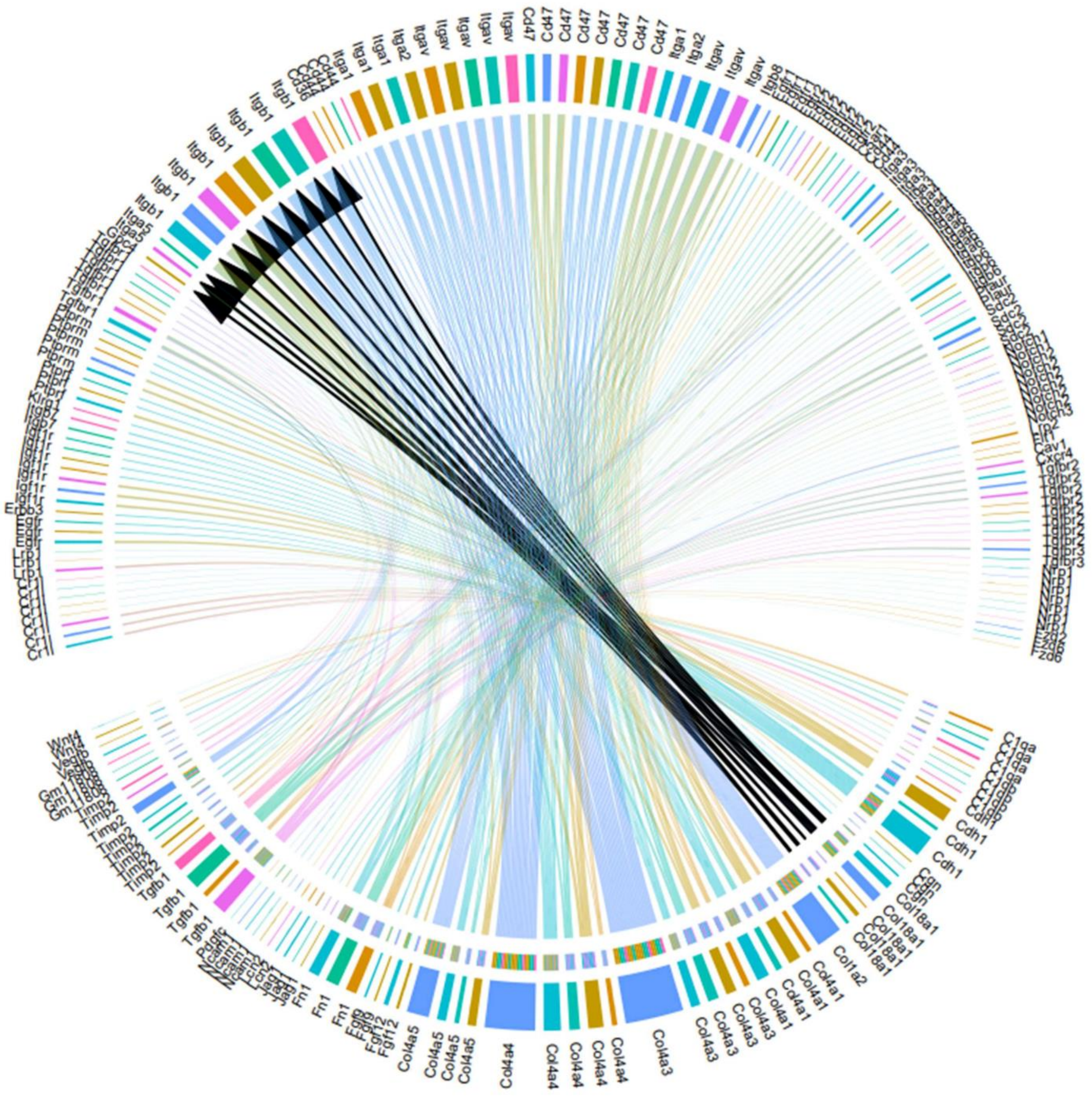

Figure S19. scRNA-seq dissects the molecular and cellular nature of distal nephron tubule segment to stromal crosstalk in the fibrotic kidney.

a

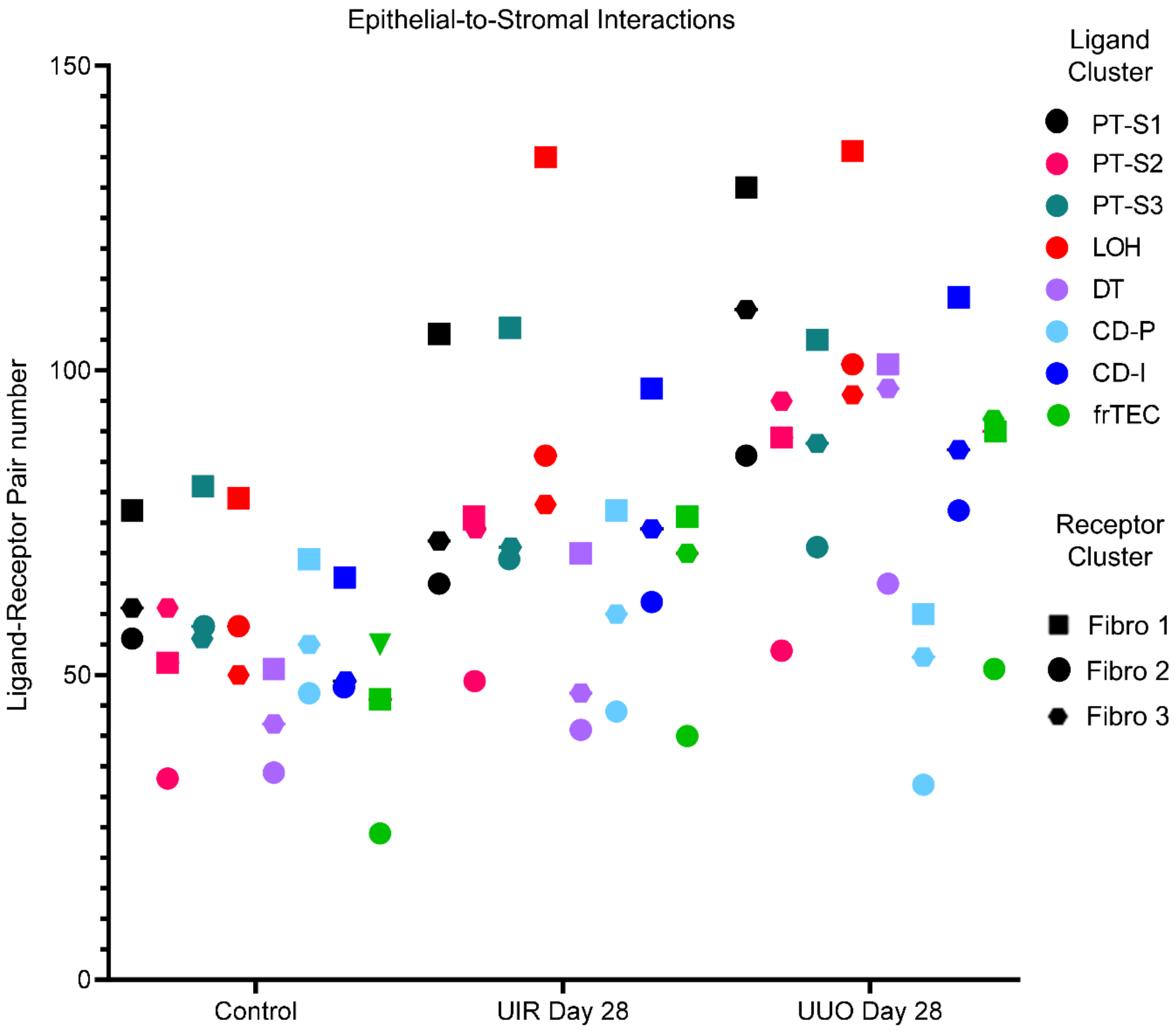

Figure S20. Quantitative analysis reveals that both kidney fibrosis models cause enhanced distal nephron tubule segment to stromal interactions. (a) Quantitative analysis of the ligand-to-receptor interaction pairs number reveals that both UIR and UUO induce elevated epithelial-to-stromal crosstalk in the injured kidneys, with the most prominent increase in loop of Henle-to-Fibroblast 1 communications. Number of interactions between the given epithelial cluster (upper right panel, color-coded) and Fibro 1 is shown with the square, Fibro 2 – with the circle, Fibro 3 – with hexagon.

a

UIR Day 28

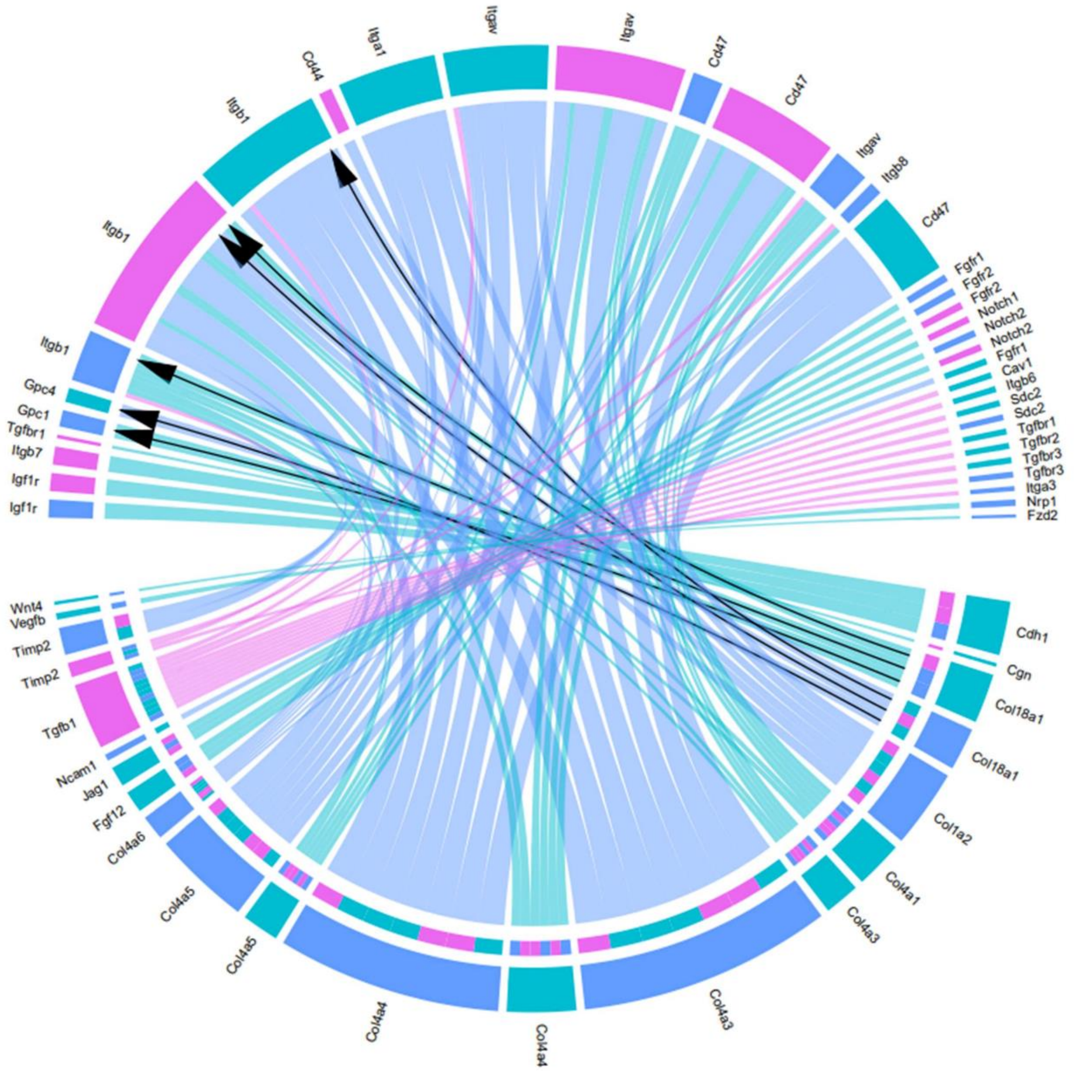

Figure S21. scRNA-seq dissects the molecular nature of the crosstalk between fibroblast clusters in the UIR induced advanced kidney injury. Circus plots display stromal-to-stromal ligand-receptor interactions in the UIR (S19a) and UUO (S20a) kidneys. Outer portion of the lower part of the circus plot represents populations expressing ligand encoding gene. Inner portion of the lower part of the circus plot as well as the upper part represents those populations expressing receptor encoding genes. Populations are color-coded identically to the UMAPs in Figure 1c. Black arrows highlight *Col18a1* and its receptor encoding genes. Complete lists of scRNA-seq predicted ligand-receptor interactions are presented in the Supplementary table S5. Note the remarkable increase in the crosstalk orchestrated by Fibro 3 via Tgfβ1 pathway.

a

UUO Day 28

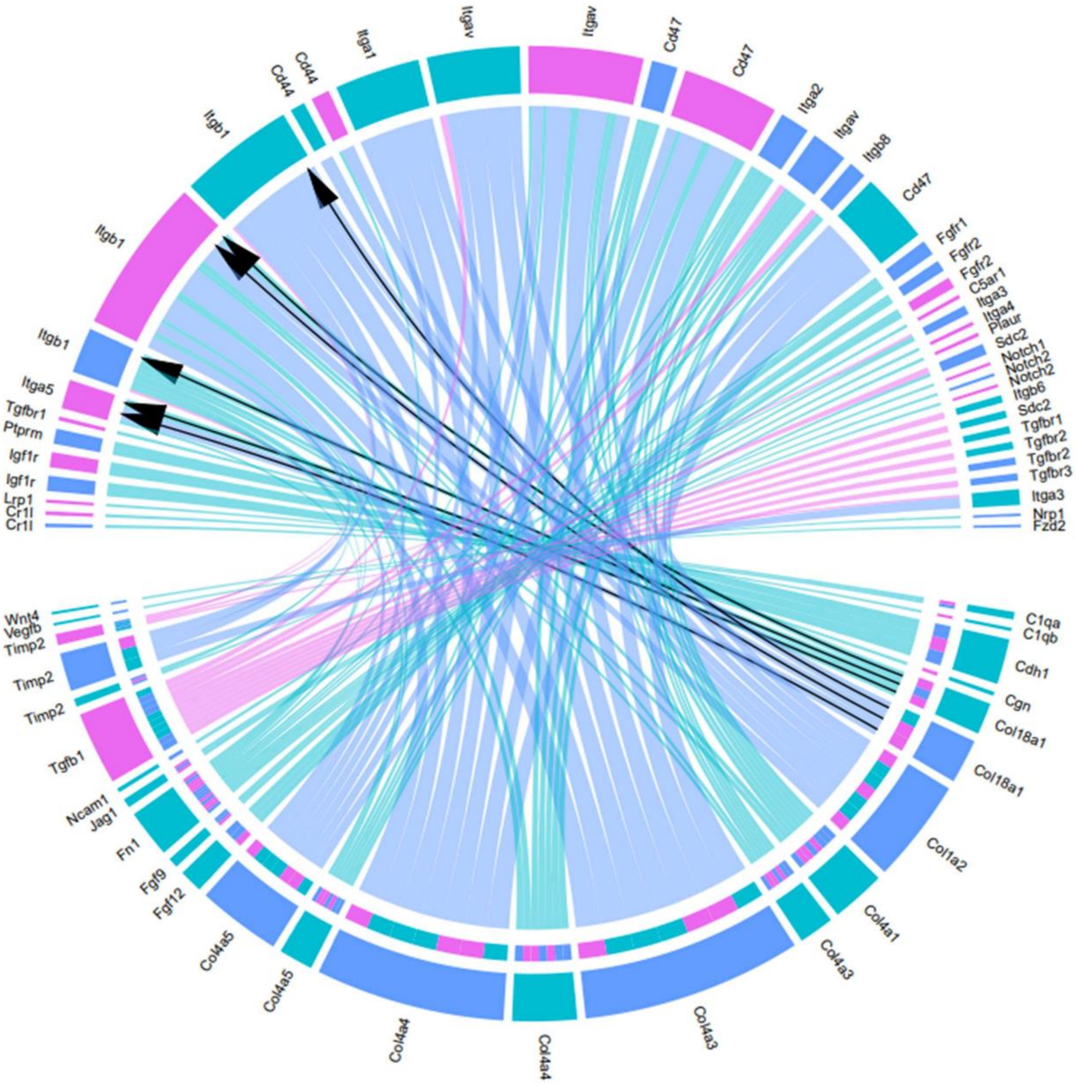

Figure S22. scRNA-seq dissects the molecular nature of the crosstalk between fibroblast clusters in the UUO induced advanced kidney injury. Circus plots display stromal-to-stromal ligand-receptor interactions in the UIR (S19a) and UUO (S20a) kidneys. Outer portion of the lower part of the circus plot represents populations expressing ligand encoding gene. Inner portion of the lower part of the circus plot as well as the upper part represents those populations expressing receptor encoding genes. Populations are color-coded identically to the UMAPs in Figure 1c. Black arrows highlight *Col18a1* and its receptor encoding genes. Complete lists of scRNA-seq predicted ligand-receptor interactions are presented in the Supplementary table S5. Note the remarkable increase in the crosstalk orchestrated by Fibro 3 via Tgfβ1 pathway.

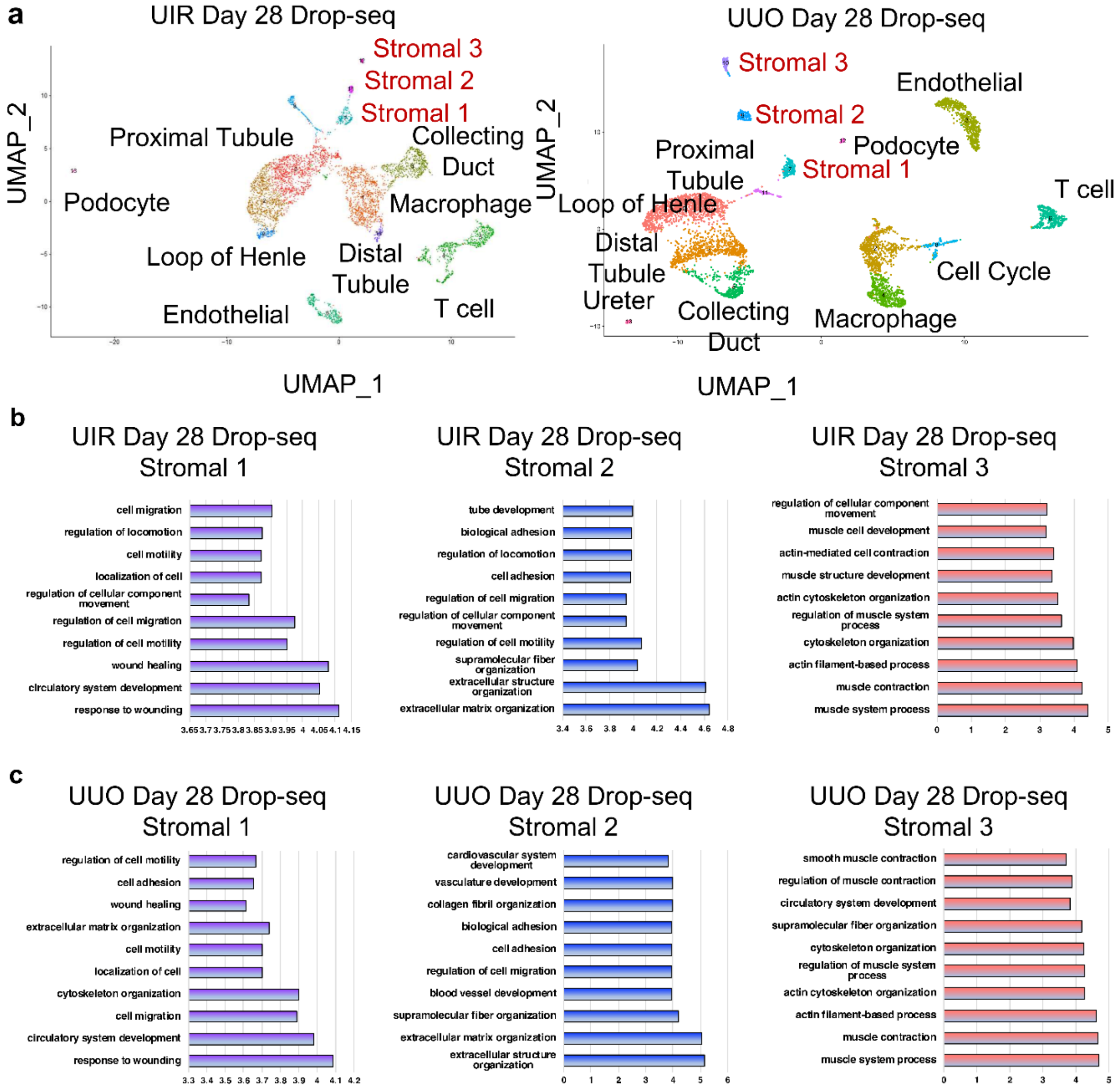

Figure S23. Drop-seq analysis corroborates 10x Chromium scRNA-seq identified three distinctive fibroblast clusters present in both UIR and UUO Day 28. (a) Feature plots demonstrating tubular, endothelial, podocyte, immune and three distinctive stromal clusters in UIR and UUO models of kidney fibrosis. (b) GO Biological process of “Stromal 1, 2 and 3” marker genes vs other populations in UIR, – log2 (*P*). (c) GO Biological process of “Stromal 1, 2 and 3” marker genes vs other populations in UUO, – log2 (*P*).

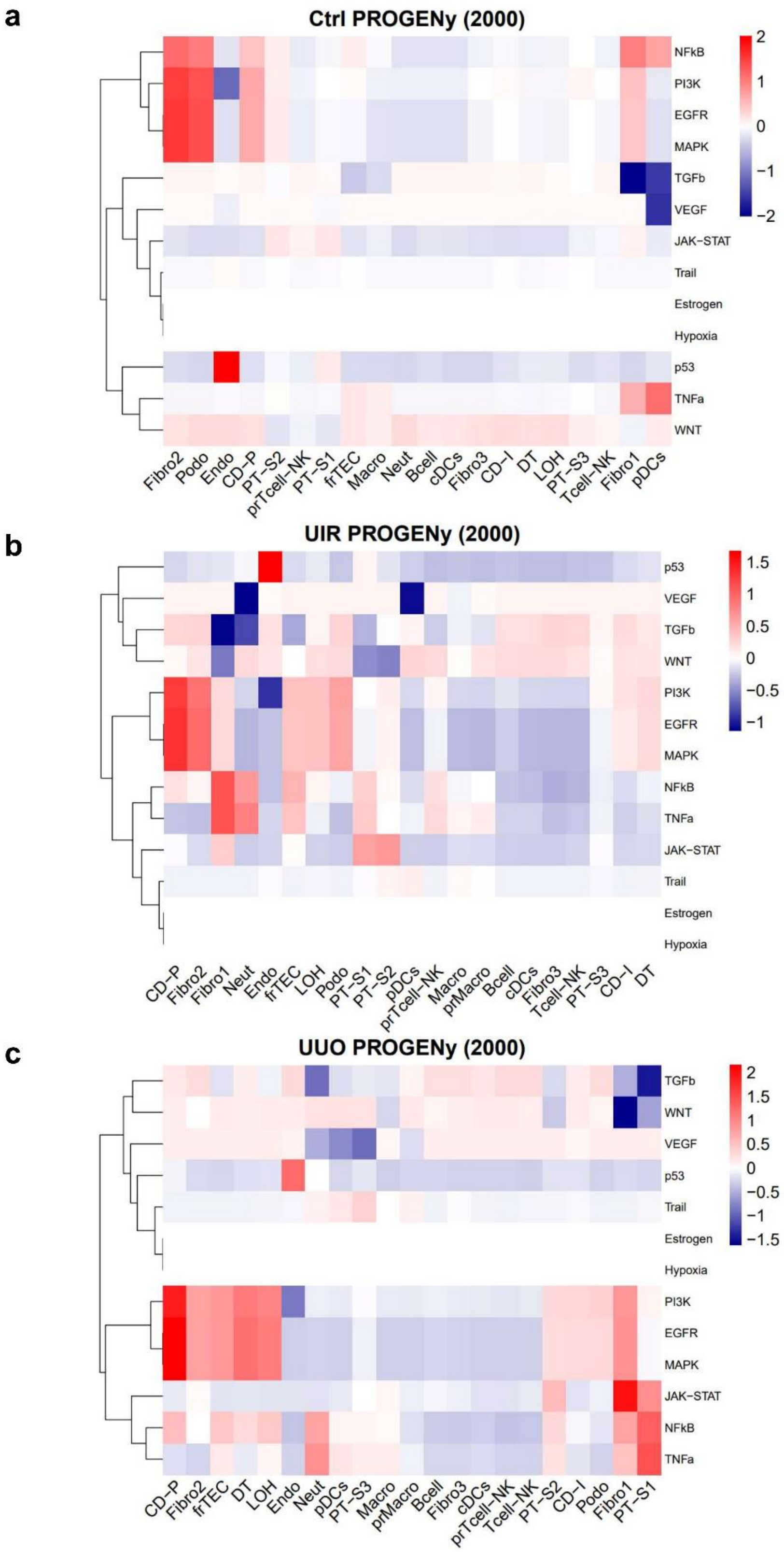

Figure S24. PROGENy analysis reveals the molecular signaling pathway changes caused in the kidney cell populations by long-term fibrotic injuries. (a-c) PROGENy analysis of the molecular pathways enriched in the control, UIR Day 28 and UUO Day 28 kidney cell populations. Expression levels are represented with color gradient.

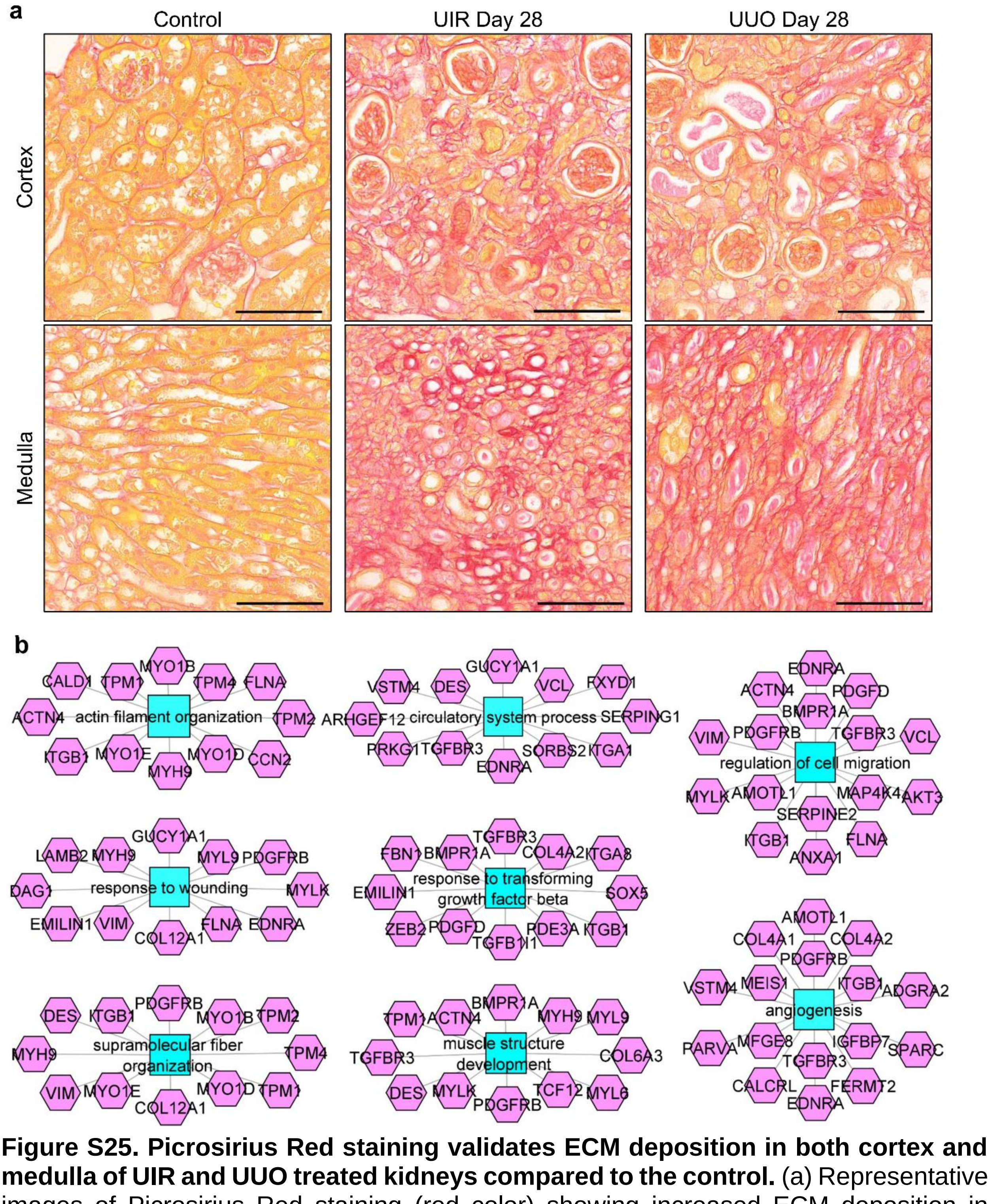

Figure S25. Picrosirius Red staining validates ECM deposition in both cortex and medulla of UIR and UUO treated kidneys compared to the control. (a) Representative images of Picrosirius Red staining (red color) showing increased ECM deposition in cortex (upper) and medulla (lower) of fibrotic kidneys compared to control. Original magnification, ×40, 0.16 µm/px zoom. (b) ToppCluster analysis of biological processes enriched among 243 shared Fibro 1, 2 and 3 marker genes. Biological processes are highlighted with blue polygons, individual gene names – pink polygons. A complete list of genes is available in the Supplementary table S4.

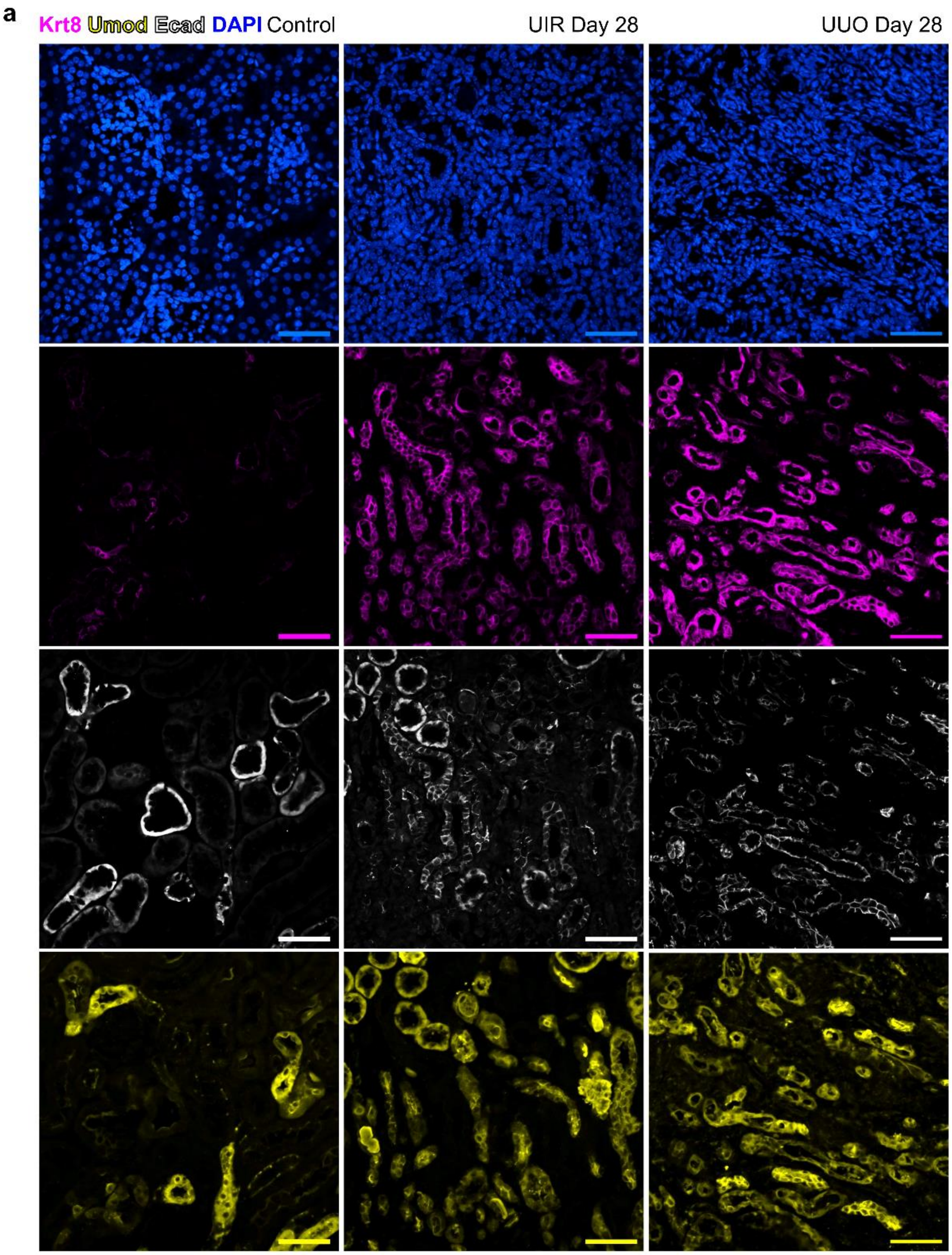

Figure S26. Long-term kidney parenchymal remodeling exhibits distal spatial pattern of tubular injury. (a) Single channels for combined IF for Krt8 (magenta), Umod (yellow), Ecad (white) and DAPI (blue) in control, UIR and UUO kidneys. Original magnification, maximal intensity projection, ×60, 0.14 μm/px zoom.

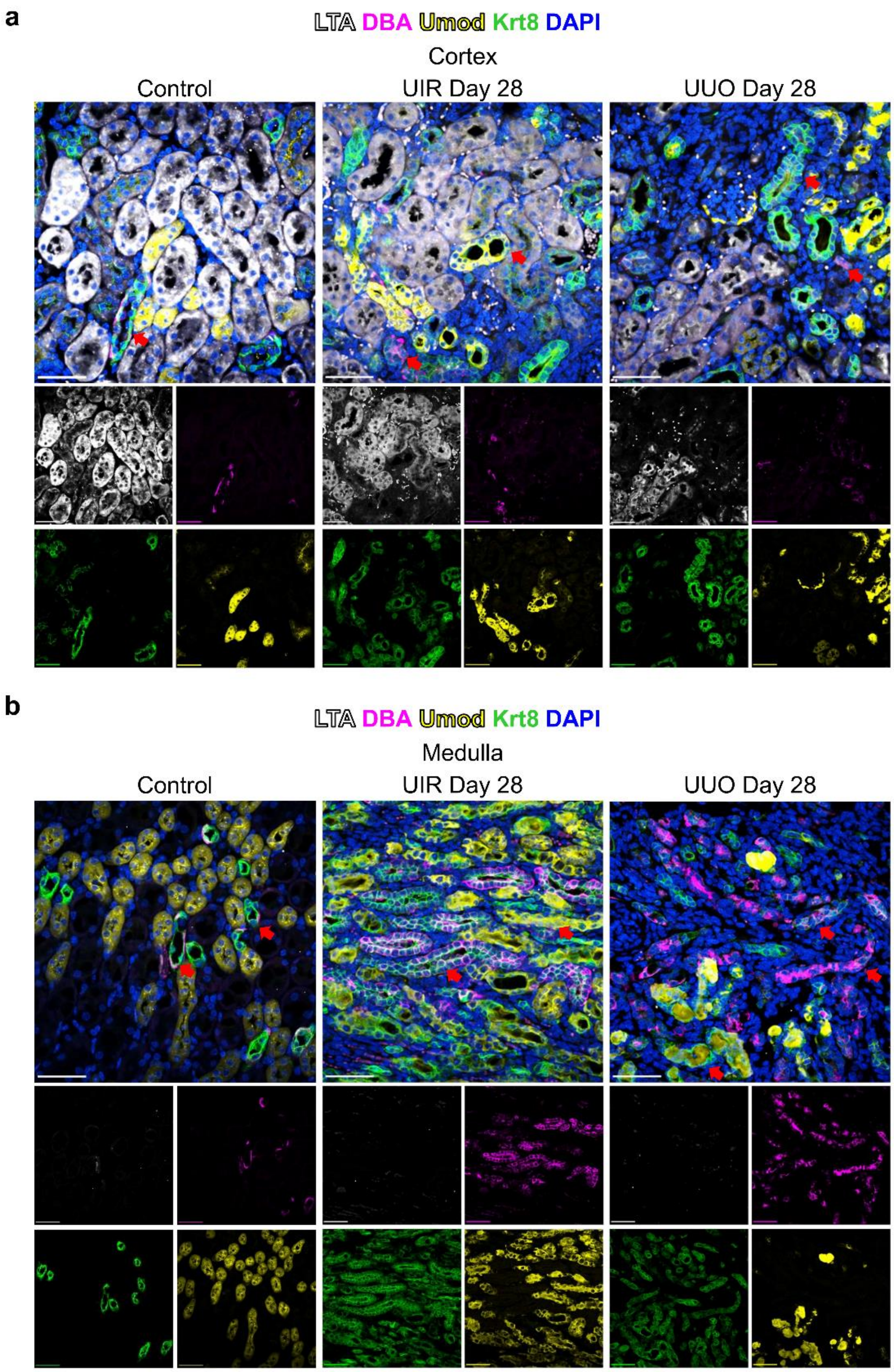

Figure S27. Krt8 expression spares LTL-positive proximal tubules and overlaps with distal nephron tubular segments. Representative images of combined IF for LTL, (white), Umod (yellow), DBA (magenta), Krt8 (green) and DAPI (blue) in the control, UIR and UUO kidneys. Original magnification, ×60, maximal intensity projection, 0.14 μm/px zoom.

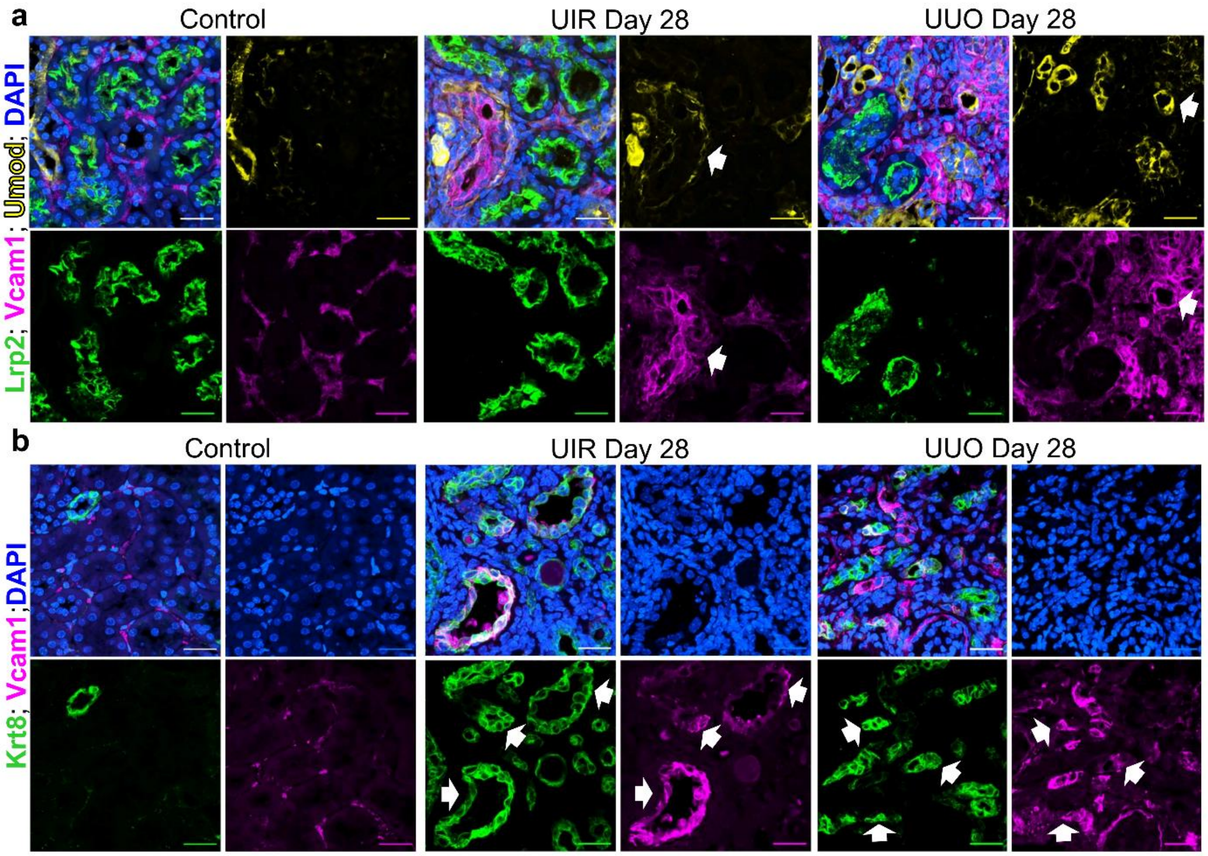

Figure S28. UIR and UUO induced kidney fibrosis caused intratubular Vcam1 expression in Umod- and Krt8-positive tubules. (a) Representative images of combined IF for Lrp2 (green), Umod (yellow), Vcam1 (magenta) and DAPI (blue) in the control, UIR and UUO kidneys. Original magnification, ×60, maximal intensity projection, 0.14 μm/px zoom. White arrows highlight Vcam1 and Umod colocalization. (b) Representative images of combined IF for Krt8 (green), Vcam1 (magenta) and DAPI (blue) in the control, UIR and UUO kidneys. Original magnification, ×60, maximal intensity projection, 0.14 μm/px zoom. White arrows highlight Vcam1 and Krt8 colocalization.

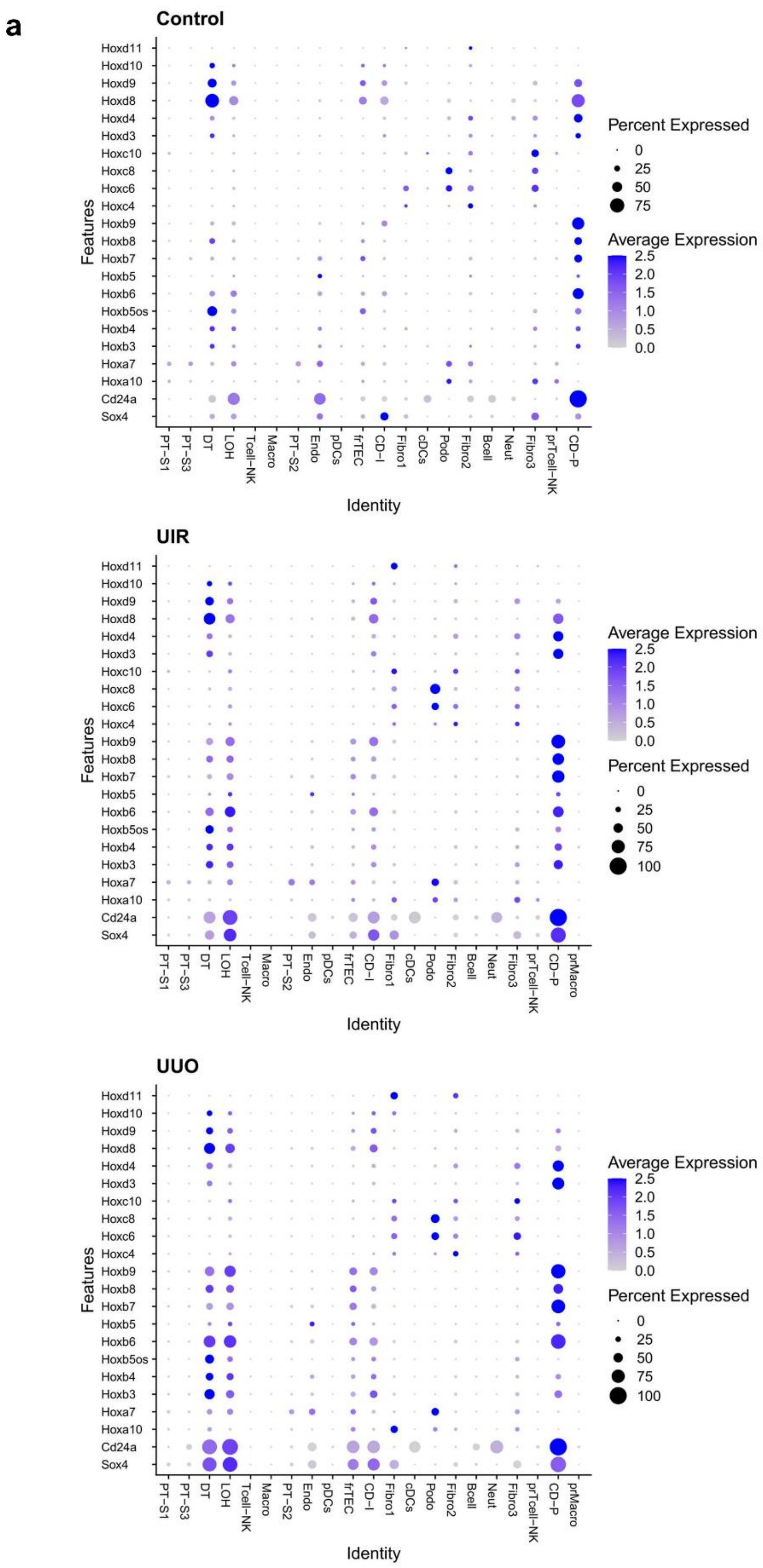

Figure S29. Advanced fibrotic injuries cause renal developmental program reactivation in the distal nephron tubular segments of adult kidney.

(a) Dot plot of cell type-specific expression of renal developmental genes for manually annotated clusters in the control, UIR and UUO kidney. Dot size denotes percentage of cells expressing the marker. Color intensity represents average gene expression values.

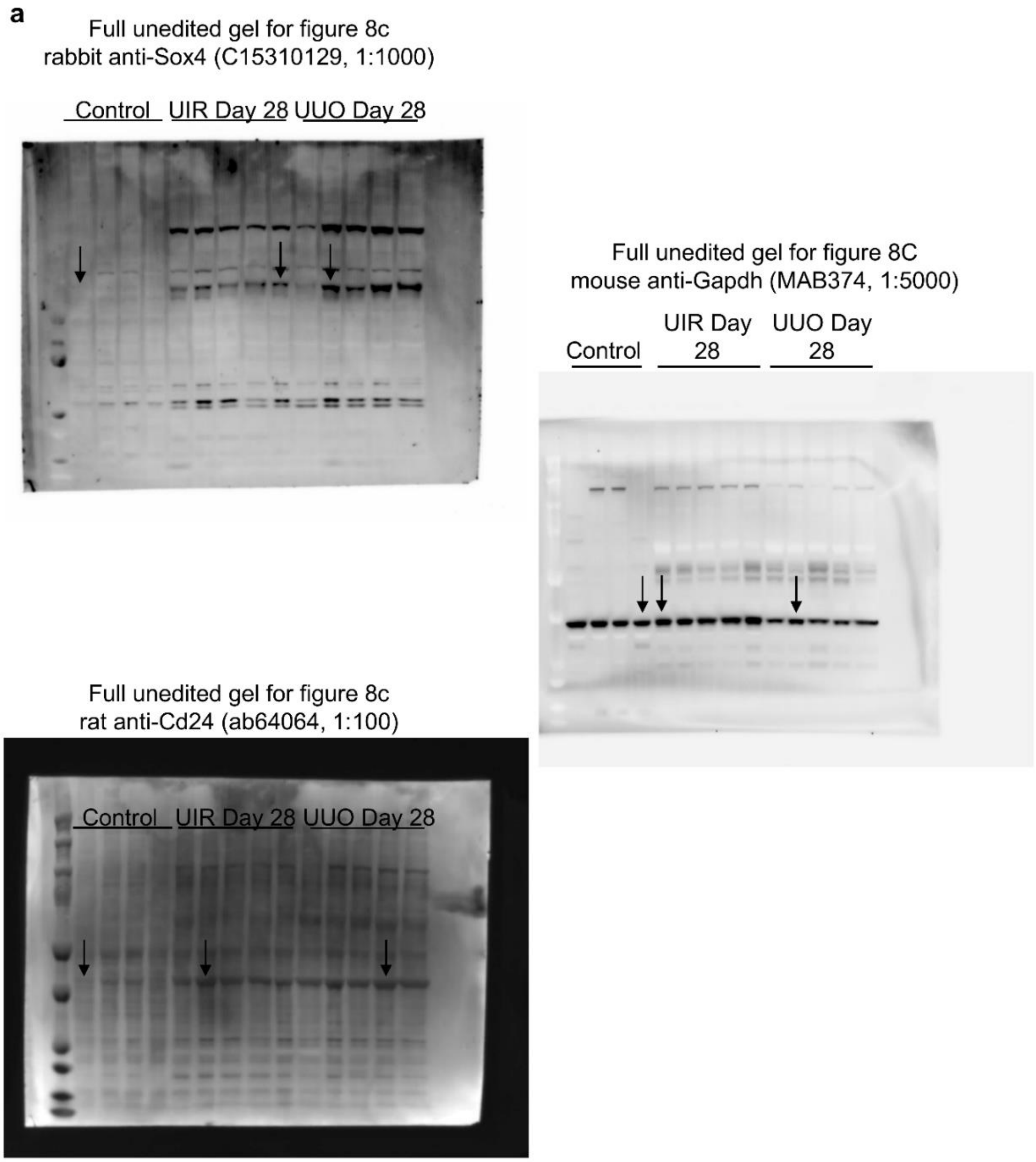

Figure S30. Original uncropped blots for Figure 8c. (a) Original uncropped images of Western blots for Sox4 and Cd24a along with the positive control (Gapdh), n=4-5 per group. Representative bands shown in figure 8c are highlighted with black arrows.
